# The development and deployment of Common Data Elements for tissue banks for translational research in cancer – An emerging standard based approach for the Mesothelioma Virtual Tissue Bank

**DOI:** 10.1186/1471-2407-8-91

**Published:** 2008-04-08

**Authors:** Sambit K Mohanty, Amita T Mistry, Waqas Amin, Anil V Parwani, Andrew K Pople, Linda Schmandt, Sharon B Winters, Erin Milliken, Paula Kim, Nancy B Whelan, Ghada Farhat, Jonathan Melamed, Emanuela Taioli, Rajiv Dhir, Harvey I Pass, Michael J Becich

**Affiliations:** 1 Department of Biomedical Informatics, University of Pittsburgh School of Medicine, Pittsburgh, PA, USA; 2 Department of Pathology, University of Pittsburgh School of Medicine, Pittsburgh, PA, USA; 3 Translating Research Across Communities, USA; 4 Department of Epidemiology, University of Pittsburgh School of Medicine, Pittsburgh, PA, USA; 5 Department of Pathology, New York University School of Medicine, New York, NY, USA; 6 Department of Cardiothoracic Surgery, Division of Thoracic Surgery and Thoracic Oncology, New York University School of Medicine, New York, NY, USA

## Abstract

**Background:**

Recent advances in genomics, proteomics, and the increasing demands for biomarker validation studies have catalyzed changes in the landscape of cancer research, fueling the development of tissue banks for translational research. A result of this transformation is the need for sufficient quantities of clinically annotated and well-characterized biospecimens to support the growing needs of the cancer research community. Clinical annotation allows samples to be better matched to the research question at hand and ensures that experimental results are better understood and can be verified. To facilitate and standardize such annotation in bio-repositories, we have combined three accepted and complementary sets of data standards: the College of American Pathologists (CAP) Cancer Checklists, the protocols recommended by the Association of Directors of Anatomic and Surgical Pathology (ADASP) for pathology data, and the North American Association of Central Cancer Registry (NAACCR) elements for epidemiology, therapy and follow-up data. Combining these approaches creates a set of International Standards Organization (ISO) – compliant Common Data Elements (CDEs) for the mesothelioma tissue banking initiative supported by the National Institute for Occupational Safety and Health (NIOSH) of the Center for Disease Control and Prevention (CDC).

**Methods:**

The purpose of the project is to develop a core set of data elements for annotating mesothelioma specimens, following standards established by the CAP checklist, ADASP cancer protocols, and the NAACCR elements. We have associated these elements with modeling architecture to enhance both syntactic and semantic interoperability. The system has a Java-based multi-tiered architecture based on Unified Modeling Language (UML).

**Results:**

Common Data Elements were developed using controlled vocabulary, ontology and semantic modeling methodology. The CDEs for each case are of different types: demographic, epidemiologic data, clinical history, pathology data including block level annotation, and follow-up data including treatment, recurrence and vital status. The end result of such an effort would eventually provide an increased sample set to the researchers, and makes the system interoperable between institutions.

**Conclusion:**

The CAP, ADASP and the NAACCR elements represent widely established data elements that are utilized in many cancer centers. Herein, we have shown these representations can be combined and formalized to create a core set of annotations for banked mesothelioma specimens. Because these data elements are collected as part of the normal workflow of a medical center, data sets developed on the basis of these elements can be easily implemented and maintained.

## Background

The tremendous amount of development in molecular and systems biology driven by advancement in translational medicine and the increasing demands for biomarker validation studies have helped to catalyze the path to personalized medicine. To make these promises a reality, researchers from various basic sciences as well as clinical disciplines are realizing the need for biospecimens with high-quality clinical annotation. This need has led to the development of controlled vocabularies in order to generate a robust system of clinical annotation that will allow samples to be better matched to the research queries at hand and experimental results to be better understood and more easily verified. Additionally, this standardization allows comparative research, data-sharing and in-depth analysis of data among institutions [1,2]. The general consensus that the recommendation in the RAND Corporation's Case Studies of Existing Human Tissue Repositories, that ".... the collection of consistent and high-quality data associated with every biospecimen and employing a standardized set of common data elements [for annotation]..." is now broadly considered best practice reflects the need for such standardization [3].

The concept of tissue banking and the informatics infrastructure coupled with it has emerged as a successful strategy to support clinical and translational research. The need for controlled vocabularies in medical computing systems is widely recognized. Even systems dealing with narrative text and images provide enhanced capabilities through coding of their data using controlled vocabularies and ontology with semantic interoperability. Development of Common Data Elements (CDEs) involves the creation of a distinct readable phrase or sentence associated with a data element within a data dictionary. Utilization of CDEs then helps to describe and extract meaning or semantics from that data element. And finally, collection of CDEs in a uniform manner across multiple institutions allows data sharing in a standardized fashion [4,5].

Over the past decade, oncologists, researchers and bioinformaticians began to articulate some of these requirements by devising systems to develop CDEs with the help of ad hoc sets of controlled terms for use in their local applications. Subsequently, well-characterized data elements were created and established by the National Cancer Institutes initiatives such as the Cooperative Breast Cancer Tissue Resource (CBCTR) [6,7], Cooperative Prostate Cancer Tissue Resource (CPCTR) [8-11] and the Cancer Biomedical Informatics Grid (caBIG) [12]. In a statewide effort, we have accomplished this standardization in the Pennsylvania Cancer Alliance Bioinformatics Consortium (PCABC) [13].

In April of 2006 the National Institute for Occupational Safety and Health (NIOSH) of the Center for Disease Control and Prevention (CDC) recognized the need for an effort to create a repository of human mesothelioma biospecimen. It issued an RFA to collect large numbers of fresh as well as archival pleural, pericardial and peritoneal mesothelioma tissue specimens along with blood and DNA samples with associated epidemiologic, pathologic, genotypic and follow-up data [14]. In September 2006, the University of Pittsburgh School of Medicine was awarded funds to develop a national mesothelioma tissue bank (Mesothelioma Virtual Bank for Translational Research or MVB) (Grant #: 1U19OH009077-01) [15]. The primary goal of this initiative is to integrate multimodal data from various clinical, pathologic and molecular systems into an architecture which is supported by a set of common data elements in order to facilitate basic science, clinical and translational research. These systems are designed to facilitate semantic and syntactic interoperability in the development of data elements using controlled vocabulary and ontology in order to make the data understandable and shareable for end-users. During the initial phase of this project, the basic components and standards developed by CBCTR, CPCTR, PCABC, and cancer Data Standard Repository (caDSR) model of caBIG were utilized [6-13,16].

The process of developing CDEs typically engages many individuals and it can take several months to reach consensus among those involved. In the case of MVB a group of thoracic surgeons, mesothelioma-focused translational researchers, oncologists, thoracic pathologists, epidemiologists, cancer registrars, and data managers from various centers as well as our MVB Research Evaluation Panel (REP) provided input and approved changes in the CDEs as they were developed. In this process it was essential to 1) include experts from multiple disciplines, 2) consider the works of others who had created similar CDEs, and 3) consider established standards when available. Herein we describe the process of developing the CDEs and believe this model is scalable to other organ specific tissue banking efforts for translational research.

## Methods

### Participating Institution

Current participating institutions in the MVB include the Universities of Pennsylvania, Pittsburgh and New York University (NYU). Also supporting this effort are the Mesothelioma Applied Research Foundation (MARF), and a newly awarded P01 grant to Dr. Michele Carbone (University of Hawaii) that includes members of the MVBs REP: Dr. Brooke Mossman (University of Vermont), Dr. Joseph Testa (Fox Chase Cancer Center) and Dr. Harvey Pass (NYU). Other participants will be added to the consortium as the program expands.

### Honest Broker Concept and Human Subject Protection

The MVB uses decentralized sample and data collection and storage. Every case is assigned a de-identified MVB number. All specimens are collected using a standardized Institutional Review Board (IRB)-approved protocol (Please refer to Additional file 1) and measures are taken to ensure that proper confidentiality and privacy of human subjects is maintained with approval by the Institutional Review Board (IRB) of each participating institution. The only linkage to the patient identity is retained locally within the institution, and there are no links connecting records to patients. In addition, queries of publicly available websites generate de-identified datasets for the research community [the so-called "safe harbor" approach to HIPPA (the Health Insurance Portability and Accountability Act)-compliance] [17]. Age range rather than specific dates, are provided instead of the dates of birth and of diagnosis to meet HIPPA compliant requirements and still provide adequate data for most research purposes. Additionally, the Honest Broker Service developed by the University of Pittsburgh ensures compliance of the MVB with specific regulatory agency guidelines for the release of Protected Health Information (PHI), including those of the Office of Human Research Protection (OHRP) of the Department of Health and Human Services (HHS), HIPAA, and the University of Pittsburgh Medical Center (UPMC)/University of Pittsburgh Institutional Review Board (IRB) [17]. UPMC and the University of Pittsburgh facilitated the process for developing honest broker services, and the Department of Biomedical Informatics took the initiative in developing the first, cross-divisional, collaborative broker service. IRB approval for the UPMC/University of Pittsburgh honest broker service was attained on May 8, 2003 (IRB Approval # HB015) (Please refer to Additional files 2 and 3) [18]. Oversight for the service is provided by the Director of the Registry Research Information Service, and all honest brokers are certified in accordance with UPMC and University of Pittsburgh IRB policy. HB015 is used as the "gold-standard" model by the UPMC/University of Pittsburgh IRB.

Various types of data requests are generated, including those for hospital operational statistics (clinical and quality improvement, marketing, process improvement, and incidence reports), research preparation (review of PHI or aggregate data only for the purpose of preparing a research hypothesis and protocol), and non-human subjects research (whereby all PHIs are de-identified prior to use by the researcher), and research involving human subjects (requires full-IRB approval). The exempt research classification designates circumstances deemed by the IRB to be excused from research authorization, such as the use of de-identified health information or limited data sets (modified "Safe Harbor" permitting state, city, full zip code, and dates) provided by a certified honest broker, and research limited to deceased patients.

All data requests are tracked in a secure, web-based data request tool, regardless of whether the purpose is clinical or research-related. This tool provides the capability of measuring trends in Registry use and other data sources, and serves as the mechanism for assuring compliance with IRB policies through tracking of broker certification and IRB-approved/exempt projects. De-identification of PHI can be done manually by the honest broker by stripping the HIPAA-identified PHI and replacing with a unique code for each case, or through use of automated de-identification applications. Unique codes associated with each record must be maintained on a project-specific basis by the designated honest brokers. Full de-identification of electronic text-based documents for research projects are referred to the Center for Clinical Research Information Services (CRIS).

Clinical Research Information Services is a jointly sponsored service of the University of Pittsburgh Office of Clinical Research and the Department for Biomedical Informatics, and a certified honest broker with the University of Pittsburgh IRB possessing a business associate agreement with UPMC (our health system). CRIS uses the De-ID^© ^application developed by the Department for Biomedical Informatics at the University of Pittsburgh and licensed by the University of Pittsburgh to De-ID Data Corp, Philadelphia, PA. The De-ID application is also used by the National Cancer Institute and other academic medical centers for various research applications. The application uses a set of heuristics to identify the presence of any of the HIPAA-specific identifiers within electronically stored medical text. Though configurable for either Safe-Harbor or Limited Data Sets, the disadvantage of applying De-ID^© ^is the removal of a small amount of clinical information during the de-identification process. A linkage file stored in an encrypted format is created when a dataset is processed. The study identifier is a two-part code: part one is the number of the report for that patient, and part two is a unique 12 alphanumeric code for that patient. The major advantage of using De-ID^© ^is that the study id remains consistent across data sets and different admissions and/or multiple reports can be easily identified. In addition, the output generated by De-ID^© ^is briefly reviewed prior to releasing it to the investigator.

### Organization of the Resource

**Figure 1:**
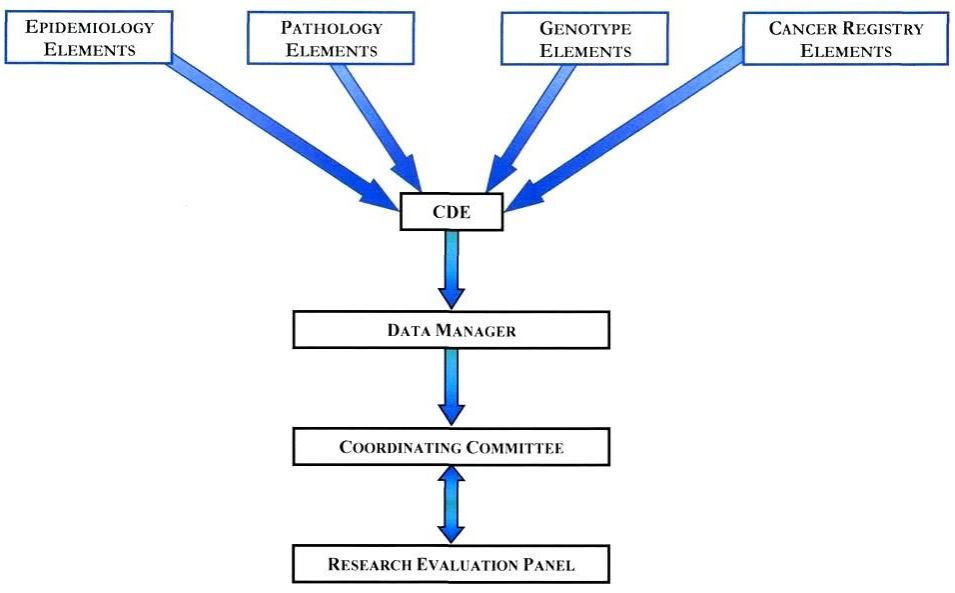
**MVB Organization of the Resource**. The Coordinating Committee determines the types of biospecimens in the repository. The Research Evaluation Panel (REP) is the committee in consultation with the Coordinating Committee. The sub-committees for Pathology, Epidemiology, Genotype (Molecular), Cancer Registry data and data managers coordinate each other to develop the CDEs for different types of biospecimens that MVB has collected.

The MVB is governed by a Coordinating Committee that has handed over responsibilities to several subcommittees (pathology, epidemiology and cancer registry). Various sub-committees that are involved in the CDE development are illustrated in Figure 1. The Coordinating Committee includes the principal investigators (PIs), members of the REP, patient advocates, database coordinators, and data managers that review request for tissues and data by end-users. The Committee's main function is to manage and supervise all of the activities of the MVB. In addition, the Committee's role in developing the CDEs was to determine the types of biospecimens (i.e. paraffin archival tissue, tissue microarrays, frozen tissue, etc.) the biospecimen resource will provide.

The major role of the pathology and epidemiology subcommittees was to develop standard evaluation guidelines and propose pathology-, epidemiology-, and genotype-specific CDEs related to the different types of biospecimens collected. Similarly, the cancer registry team is involved in evaluation of follow-up and outcome-related CDEs.

The data manager sub-committee includes data managers from each of three member institutions and database coordinators. This sub-committee's main role is to implement and evaluate the CDEs, perform quality assurance checks on the data collected at each member institution, and to help coordinate the distribution of tissue requests and the associated data sets.

### Development of the Common Data Elements

**Figure 2:**
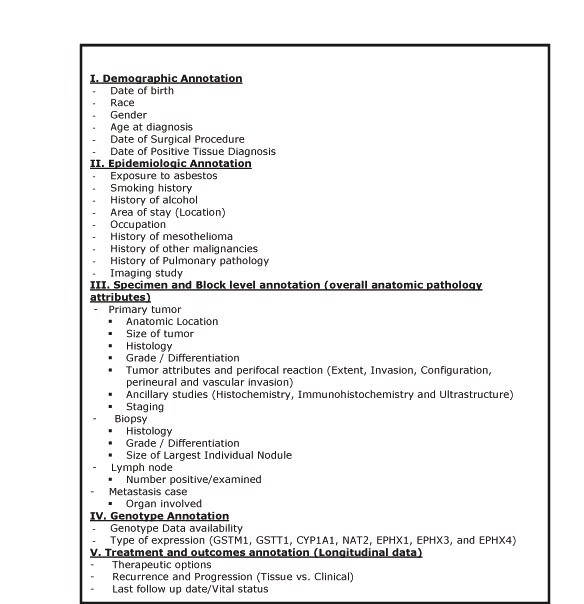
**MVB CDE categories**. Five principal categories of CDEs with their attributes and the sub-data types collected. Detail description of each CDE under these five main categories can be found in the MVB CDE data dictionary.

With leadership and supervision of the Coordinating Committee and the domain experts from the subcommittees, the CDE subcommittee was established in order to develop CDEs (demographics, epidemiology, pathology-specimen as well as block level annotation, genotype, follow-up and outcome) pertinent to pleural, pericardial, and peritoneal mesotheliomas (Figure 2). The CDE sub-committee utilized the experiences of the CBCTR [6,7], CPCTR [8-11], caBIG [12], PCABC [13], Cooperative Human Tissue Network (CHTN) [19] Early Diagnosis Research Network (EDRN) [20], Cancer Family Registries (CFR) [21], and Specialized Programs of Research Excellence (SPOREs) [22] when developing the CDEs. As stated earlier, the major standards used to formulate the CDEs include the NAACCR Data Standards for Cancer Registries [23], CAP Cancer Protocol and Checklist [24], the ADASP [25] and the American Joint Committee on Cancer (AJCC) Cancer Staging Manual [26]. Usage of these data standards, protocols, checklists and guidelines to develop CDEs provides a common conceptual model that already exists between multiple otherwise non-interoperable systems. The existence of such a conceptual model/framework is sufficient to achieve both syntactic and semantic interoperability in CDE development.

### NAACCR and UPMC Network Registry Network

The core elements and data standards set forth by the North American Association of Central Cancer Registry (NAACCR) were utilized to devise the demographic, epidemiologic, therapeutic, and outcome-related CDEs as well as easing the eventual data acquisition for the Bank. The NAACCR was first established in 1987 as a collaborative umbrella organization for cancer registries, governmental agencies, professional associations, and private groups in North America interested in enhancing the quality and use of cancer registry data [22]. It is a professional organization that develops and promotes uniform data standards for cancer registration; provides education and training; certifies population-based registries; aggregates and publishes data from central cancer registries; and promotes the use of cancer surveillance data and systems for cancer control and epidemiologic research, public health programs, and patient care to reduce the burden of cancer in North America. All central cancer registries in the United States and Canada are members. Additionally, it works to achieve consensus on cancer registration standards among the many standard setters in the United States and Canada. These include the American College of Surgeons (ACoS), the National Cancer Institute (NCI), and the Canadian Cancer Registry (CCR) at Statistics Canada.

Today nearly all registries throughout the United States and Canada have adopted the NAACCR consensus standards. These standards are updated annually. Maintaining current standards to meet the needs of the NAACCR community is an ongoing and major NAACCR activity. The principal goals of NAACCR include definition of data standards for cancer registration for use by central registries, hospital-based registries, and other groups in North America in order to provide a comprehensive reference to ensure uniform data collection that reduces the need for redundant coding and data recording between agencies and facilitates the collection of comparable data among groups. It also provides a resource document to help registries that are establishing or revising their databases and encourages the adoption of these standards by all parties. The NAACCR coordinates the implementation of standards to promote continuity in data collection, exchange, and analysis. Furthermore the agency helps in improving and maximizing dissemination, interpretation, and use of data.

Our Registry Information Services (RIS) is an emerging division of the UPMC Network Cancer Registry and the UPMC Cancer Centers [27]. The full-time staff is responsible for maintaining a standardized data system designed for the collection, management and analysis of specific patient information. The RIS collects data about demographics, medical history, diagnostic findings, cancer grading, treatment, outcomes, and American Joint Commission on Cancer (AJCC TNM) and Surveillance, Epidemiology and End Results (SEER) cancer staging. This information is used for administrative planning, education, results reporting and research for the University of Pittsburgh Cancer Institute and UPMC Cancer Centers [27]. Two distinct sub-divisions operate within the 12 hospital-based registries, 5 stand-alone clinics, and 28 physician offices comprising the UPMC Network Cancer Registry. One handles data collection and dissemination for clinical and hospital operational purposes, while the other is dedicated to the specific efforts and data needs of the research community of the Centers of Excellence within the UPMC Cancer Centers and University of Pittsburgh Cancer Institute (UPCI). Standardized data is captured for all reportable diagnoses in accordance with the Commonwealth of Pennsylvania's Department of Health regulations, permitting this registry process to be exempt from HIPAA patient consenting processes. Additionally, the majority of facilities within the Registry adhere to the voluntary standards set forth by the American College of Surgeons Commission on Cancer for approved cancer programs. It is also a key source for Honest Broker Services established in May 2003 to meet the needs of our oncology research environment.

**Figure 3:**
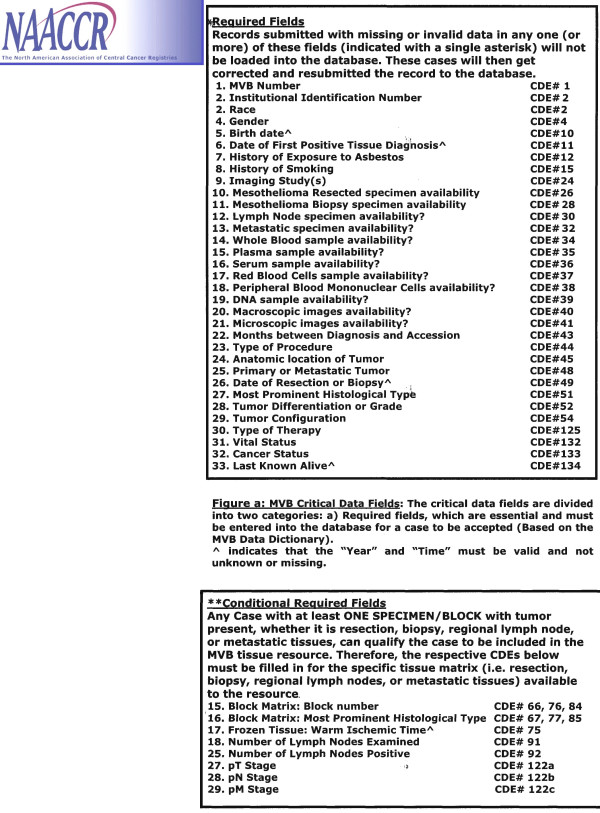
**MVB Registry Data Fields**. The critical registry data fields are divided into two categories: 1) Required fields, which are essential and must be entered into the database for a case to be accepted. 2) Conditional required fields, which must be filled out when respective biospecimen is entered into the database.

Primary sources for data extraction include both paper and electronic medical records. Data is abstracted via manual and electronic methods into the cancer registry database by Certified Cancer Registrars (Figure 3). The entire UPMC Network Cancer Registry information system is based on the architecture of the *NAACCR consensus standard*s, which were realized from standards previously set by American College of Surgeons, the National Cancer Institute, and the Canadian Cancer Registry at Statistics Canada. Updated annually, these data standards allow meaningful comparison of data across different registries, and compilation of case-specific information into useful and meaningful registries thanks to standardized case definitions, coding practices and conversions of medical terminology.

### CAP Protocol and ADASP guidelines

The core elements and data standards set forth by the College of American Pathologists (CAP) and the Association of Directors of Anatomic and Surgical Pathology (ADASP) were utilized to devise the pathology CDEs for MVB resource. In recent years, these two societies (ADASP and CAP) [25,24,28] have published guidelines for the reporting of human malignancies in order to standardize pathology reporting in an optimal way to ensure that the information necessary for diagnosis, management, prognostic and predictive factor assessment is available and understandable. The CAP assigned multidisciplinary groups of pathologists, surgeons, and radiation and medical oncologists to develop the protocols. Other pathologists and clinicians then reviewed them. After the initial reviews, the protocols were then further reviewed by multiple CAP committees and finally approved by its Board of Governors. The ADASP, in contrast, chose a pathologist expert in each field to assemble a group from within the pathology community (with clinician input if desired) to write specific cancer protocols. These were then approved by the ADASP council and subsequently by the membership. Even though both societies began the process at approximately the same time, the streamlined approach adopted by the ADASP enabled it to publish years earlier in pathology journals frequented by anatomic pathologists. Although the formats are somewhat different, the contents are essentially the same.

Recently, the American College of Surgeons Commission on Cancer (COC) decided to require elements deemed as essential by the CAP to be described in all pathology reports in accredited cancer centers as of January 2004. The COC accredits cancer centers in the United States. It is important to note that COC does not require that the specific CAP protocols or synoptic reports be used. ADASP has updated all of its protocols to comply with the COC requirements in the form of 37 uniform checklists, whereas CAP cancer checklists consist of a series of reporting guidelines for diagnostic surgical pathology reports for 45 important human cancers. The checklists use the staging criteria cited in the American Joint Committee on Cancer staging manual [25] but include a variety of other references listed in each of the checklists. Moreover, the checklists are formatted for ease of use. They may be used as templates for uniform reporting and are designed to be compatible with voice-activated transcription. The different elements in these revised ADASP diagnostic checklists have been divided into *required *and *optional*. The term *required *in this context only signifies compliance with the COC guidelines. ADASP realizes that specimens and practices vary and that it will not be possible to report these elements in every case. However, ADASP hopes that pathologists will find these checklists useful in daily clinical practice, while facilitating compliance with the new COC requirements. The checklists are in standard PDF file format, may be easily downloaded from the ADASP, and are not to be reproduced, altered, or used for commercial purposes without consent from ADASP [25].

**Figure 4:**
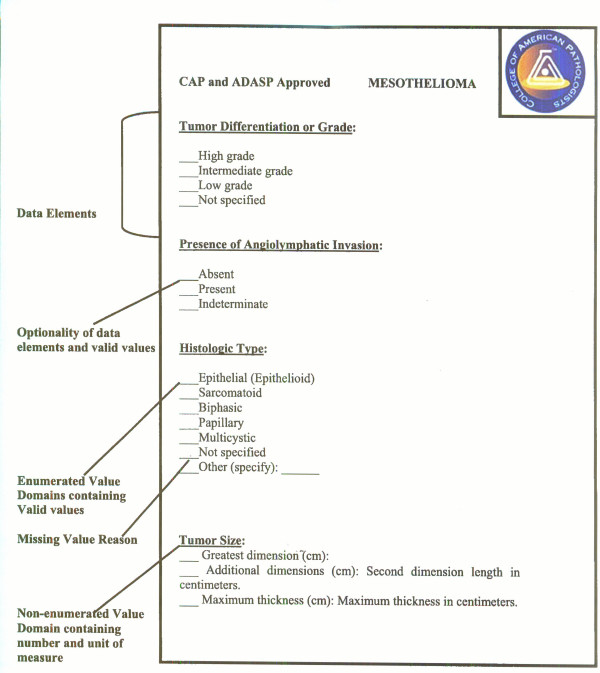
**Fragment of CAP cancer checklist and ADASP guidelines for reporting mesothelioma**. Fragment of **CAP cancer checklist and ADASP guidelines **for mesothelioma showing relationships between the Data Elements with their value domains. Checklist text reproduced with permission of the College of American Pathologists and Association of Directors of Anatomic and Surgical Pathology.

The CAP cancer protocols and checklists and ADASP guidelines are important reporting standard in pathology, a field for which no complete electronic data standard is currently available. Each guideline consists of [1] a checklist specifying the data elements of the specimen and tumor that should be included in the diagnostic pathology report as well as the valid values that these data elements may take (Figure 4), and [2] a detailed protocol providing definitions and further information about the scientific basis for assessing these variables. Each protocol and checklist was developed by a separate panel of subspecialty experts for that organ system, often representing differing schools of thought. In each case, expert panels reviewed the existing literature to determine which features provided the most important data for clinical decision-making. In addition to specifying the data elements and valid values, these guidelines provide other useful information for creating structured metadata; the paper standard [1] logically groups data elements together by surgical procedures or type of examination (macroscopic vs. microscopic), [2] distinguishes between required data elements for which there is unequivocal scientific evidence of their value, and optional data elements which do not meet that scientific threshold, and [3] maintains relevance with revised versions published as new data becomes available regarding prognostic factors and clinical outcomes. However, a common data standard which permits interchange among clinical and research systems is still urgently needed to advance tissue-based research.

### Genotype CDEs

Data related to metabolic gene polymorphisms is available for a subset of the cases. These include glutathione S-transferase M1 (GSTM1), glutathione S-transferase T1 (GSTTM1), cytochrome P450 1A1 (CYP1A1), N-acetyltransferase 2 (NAT2), and microsomal epoxide hydrolase (mEH) gene polymorphisms. The coding of genotype variants follow the standardized procedure previously described by Garte et al [29].

Usage of these data standards, protocols, checklists and guidelines to develop CDEs provide a common conceptual model that already exists between multiple non-interoperable systems. The existence of such a conceptual model is necessary to achieve either syntactic or semantic interoperability in CDE development.

### Creating information models for CDEs with the aid of Unified Modeling Language

Information models provide an abstract formal representation of the conceptual or physical entities. Information models for the MVB biorepository are constructed as UML class diagrams. The UML is a non-proprietary language for constructing, visualizing, and documenting the artifacts of software engineering. A UML class diagram is one kind of diagram that depicts a collection of static model elements such as physical or conceptual entities and their relationships [30].

**Figure 5:**
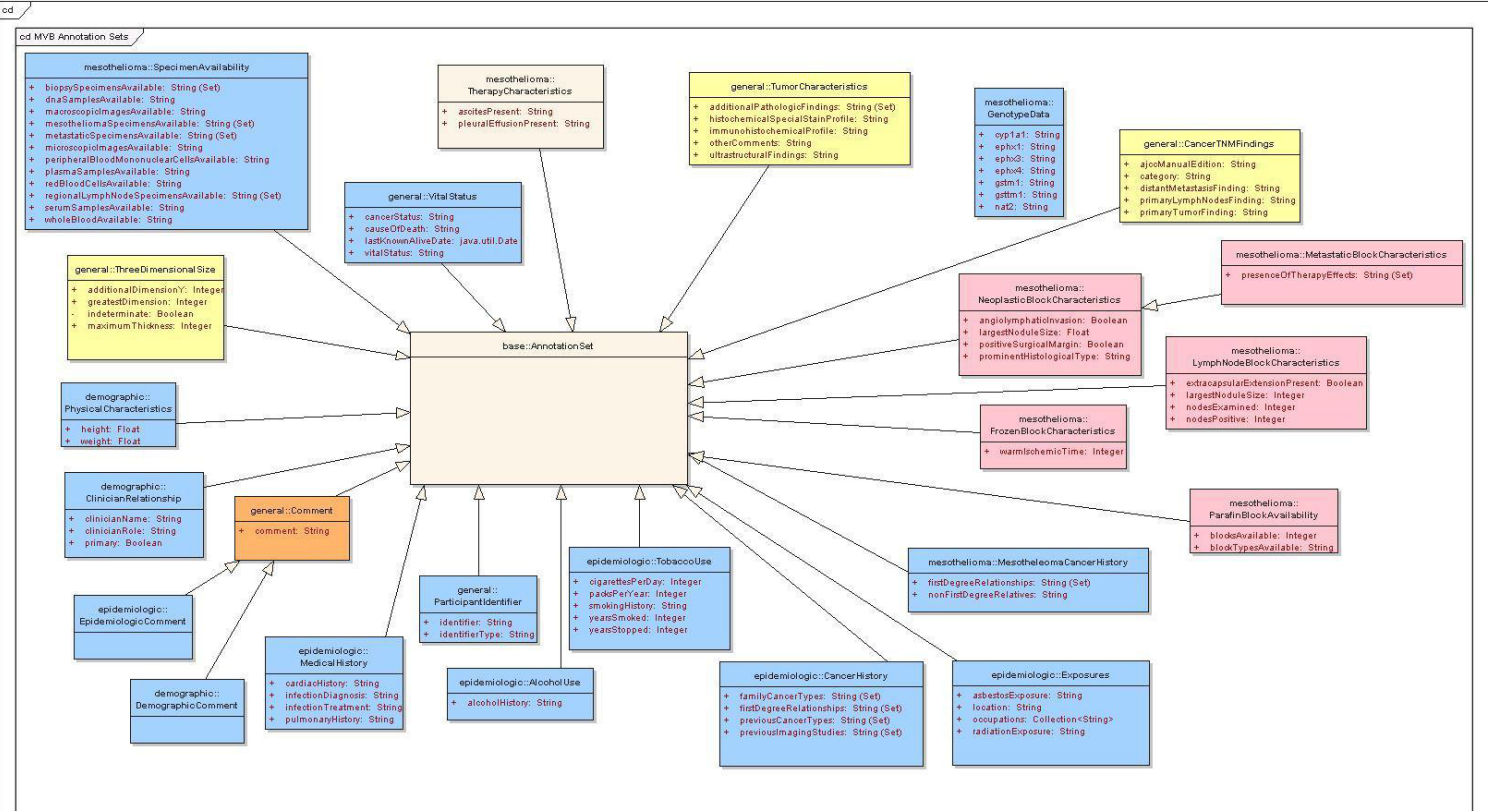
**Fragment of the UML information model for Mesothelioma**. Fragment of the CAP checklist, ADASP elements, and NAACCR elements UML class model showing association and generalization relationships of Mesothelioma

To explain, in a UML-class diagram specific conceptual entities are represented by *classes*. Each *class *may have *attributes *that describe specific characteristics of these entities. Attributes, in turn have one or multiple *values*. In many cases, permissible values within a value domain represent reasons why particular values were absent, for example because the attribute was not identified or because the attribute could not be evaluated in this context. In these cases, we segregated 'missing value reasons' from the value domain proper, creating a separate attribute name (Figure 5). Classes are related to other classes by *relationships*, which are represented by arcs between classes in the UML diagram. *Associations *are "peer-to-peer" relationships between classes. The names and cardinality of the ends of the associations are marked near the boxes that delimit the classes. Other associative relationships include *aggregations *and *compositions*, which are used to model "whole/part" relationships between classes. *Generalization *relationships model inheritance between classes. The class that is generalization of a concept is referred to as the *superclass *and the class that is specialization of a generic concept is referred to as the *subclass*. Subclasses inherit attributes and methods from their superclasses. *Enumerations *are UML stereotyped classes that provide a list of named values.

We have used Enterprise Architect (EA) as the UML modeling tool for this project due to its low cost and high performance [31]. During modeling, each UML class, attribute and enumeration value was annotated with a human readable definition of the semantic meaning of that class or attribute. These definitions were created as tagged values in UML, which provides a method for adding additional information to a UML stereotype. Models annotated with these definitions were then saved in the XML Metadata Interchange (XMI) format for further processing [32].

### Semantic metadata based on the ISO/IEC standard

Semantic interoperability requires that the meaning of the data within the system or analytic services performed by the system is unambiguous. It must be interpretable by both humans and computers. In order to achieve this – a great deal must be known about the meaning and form of the data. *Semantic metadata *provides the 'data about data' needed to interpret the meaning and form of data and to determine the relationship of one datum to another. Therefore, semantic metadata must have a common, uniform structure, and must be universally available for inspection, discovery and inference [32].

The basic representation used for semantic metadata within MVB is defined by ISO/IEC – a standard for metadata structure and registration. This specification was developed for the specific purpose of facilitating worldwide metadata standardization by providing guidance on the framework itself, the classification of data, the semantic structure of data, formulation of definitions, naming and identification, and guidance and instruction of the registration of metadata [33].

*Data elements *that conform to ISO/IEC must be associated with one *data element concept (DEC) *and one *value domain*. The DEC defines the meaning of the datum. Each DEC must have one and only one *object class *that describes the real world or conceptual entity and one and only one *property *that describes some characteristic of that entity. Both object classes and properties may also take one or more *qualifiers *that modify the meaning of the object class or property. The *value domain *represents the set of *permissible values *that are valid for this datum. Value domains are annotated with representation terms that classify the data element according to the category of data stored in the data element (e.g. indicator, code, number). Value domains may be either enumerated (e.g. as a set of valid values) or non-enumerated (e.g. as a number or character string). Data elements may be aggregated together as *classification scheme items *belonging to a *classification scheme*, which provides a method for grouping data elements into a logical hierarchical framework.

### Creating basic UML structure

High-level UML classes were then joined by relations representing the logical relationships between classes. Associative relationships were assigned directionality and cardinality. Inheritance relationships were used to extend the general classes as each specific CAP cancer checklist was developed. For example, MesotheliomaNegativeSurgicalMargin expresses the surgical margin findings specific to negative margins in cases of pleural mesothelioma. It is a subclass of the more general SurgicalMargin, and thus inherits all of the attributes of SurgicalMargin. MesotheliomaNegativeSurgicalMargin also has additional attributes that are specific to negative margins. A fragment of the total UML model is shown in Figure 5.

An important aspect of this phase of creating the model for CDEs was to determine where generalization relationships should be structurally aligned. Semantic interoperability requires unambiguous semantics. One difficulty in utilizing a multi-structure modeling environment is that apparent conflicts may arise when the structures conflict. This conflict could potentially produce significant ambiguity.

Metadata or data descriptors are then developed to describe a specific CDE. Metadata offers the potential for true interoperability between systems on two levels: syntactic interoperability, which concerns itself with the ability to exchange information; and semantic interoperability, which is the ability to understand and use the information once it is received. Metadata also has two components: attributes and valid values. Additionally, ISO/IEC standard for metadata formulation specifies that metadata should have a qualified name or identifier, an authority who registers the name, a versioning history (allowing for modifications), a language or origin, a statement relating to usage, a data typing statement, and a definition that is unambiguous [4,33]. The MVB data dictionary describing each of the common data elements were generated by following the ISO/IEC standard for metadata (Please refer to Additional files 4 and 5).

### Development of the MVB database: Informatics Architecture and Integration Issues

The MVB Project required a mechanism for making Mesothelioma cases searchable via a web interface. The University of Pittsburgh project team met this requirement using the MVB Query Tool; a web-based application that is based on the caTISSUE Clinical Annotation Engine (CAE) System [34]. The resulting system has been made available publicly on the Department of Biomedical Informatics (DBMI) website [35].

**Figure 6:**
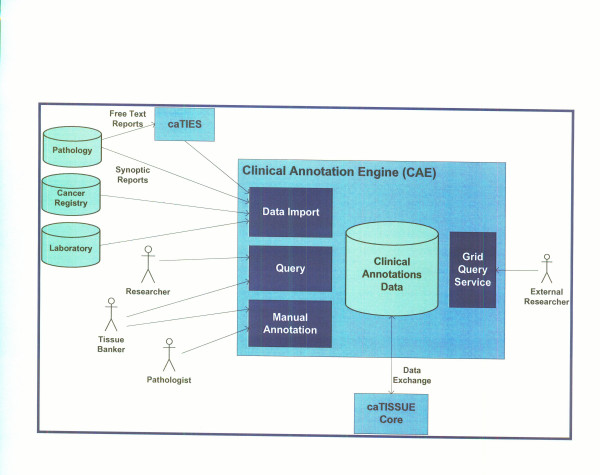
Overview of caTISSUE Clinical Annotation Engine depicting data import, integration and query interface in a web-based environment. caTIES is a tool to extract important information on archival surgical pathology report and caTISSUE core is an enterprise biospecimen repository.

caTISSUE CAE was originally developed by the University of Pittsburgh as part of the National Cancer Institute's (NCI) Cancer Biomedical Grid (caBIG) program [34]. This is a toolkit for clinical annotation of biospecimens for translational research. It is envisioned as an annotation system for the TBPT Workspace by providing standards-based annotations and tools for integrating data from existing clinical and research systems. The CAE provides for manual annotation of biospecimens with pathology, tumor marker, staging, grading and follow-up data utilizing a web-based user interface, import of structured data from clinical information systems such as Anatomic Pathology Laboratory Information Systems (APLIS), Clinical Pathology Laboratory Information Systems (CPLIS), and other specimen registries which may be employed in the life cycle of clinical trials or clinical research, and integration of annotations from multiple sources within the translational research center (Figure 6) It provides a mechanism for entering, importing and searching for biospecimen annotation. In the latest released version of this software, clinical annotations are attached to either a participant/patient, a tissue accession or to a specimen (part) or sub-specimen (block). Taken together these entities form a hierarchy or backbone that encapsulates all of the annotation data for a case. Annotations can be entered manually using the provided user interface or imported using an XML format.

The CAE system is based on a model-driven architecture. A UML class model is created using a UML model. The resulting model is used to generate the source code for domain classes and the required metadata. The annotation engine uses the domain classes and metadata to render data entry, query and result screens and to specify the XML import formats. The latest version of the CAE software includes models for eight different types of cancers: breast, prostate, melanoma, kidney, lung, pancreas, colon, rectum and central nervous system tumors and is available.

**Figure 7:**
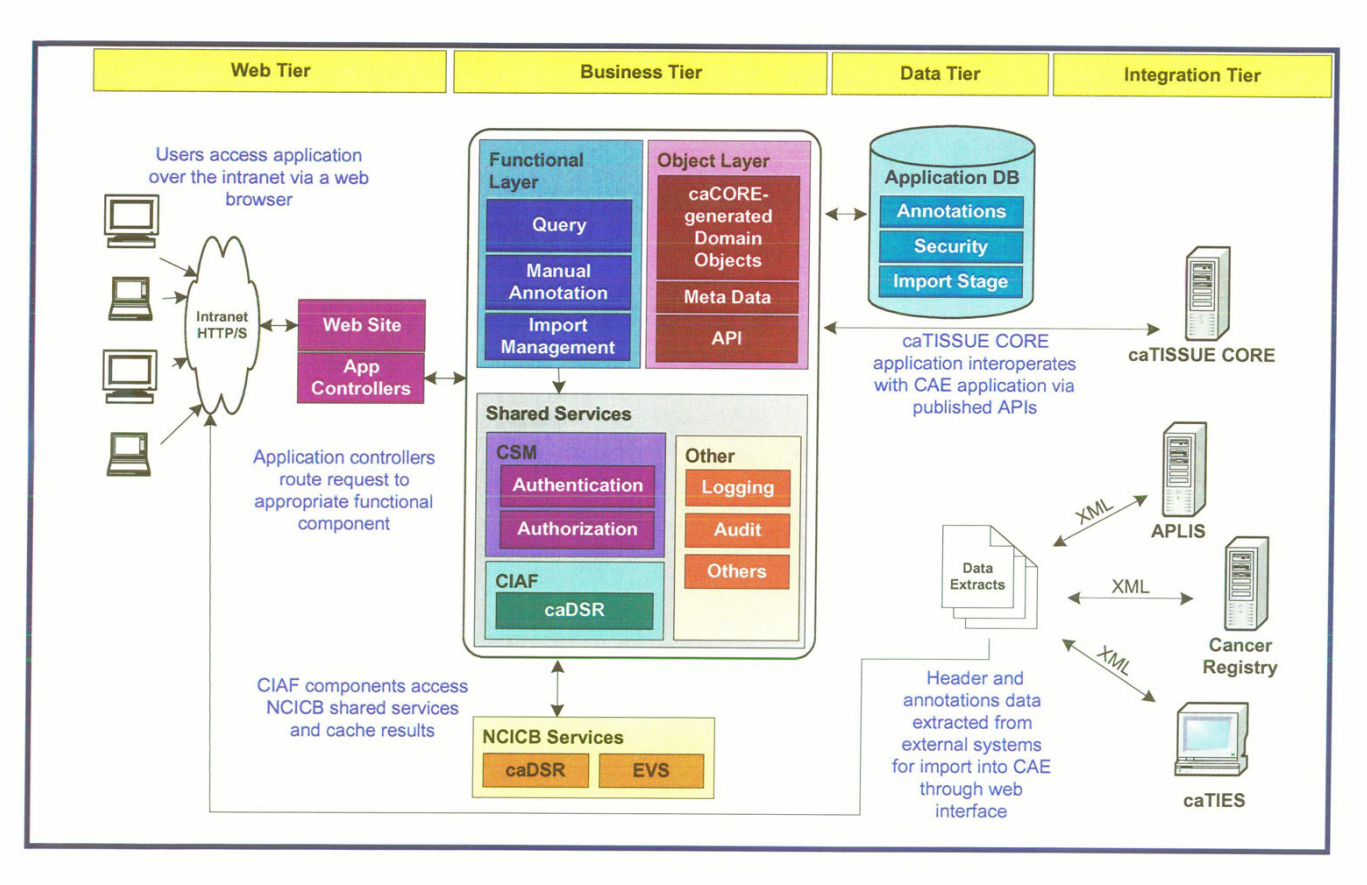
The overall architecture and tiers of caTISSUE Clinical Annotation Engine.

The various components of CAE include web-tier, business tier, data tier and integration tier (Figure 7). The web tier serves up static HTML, images, style sheets and dynamically generated web pages via a standard JSP/Servlet engine. Dynamic requests will be managed through a Model-View-Controller (MVC) mechanism. This mechanism manages the processing of individual requests and the flow between requests. The Spring Framework is being evaluated for this purpose. The controller object makes requests to the business tier that results in the return of model objects that represent the information that must be presented back to the user. Based upon the result of the controller actions, the model objects are forwarded to an appropriate view (JSP) which renders them into a displayable page. The business tier consists of a set of functional components, an Object-Relational (O/R) mapping mechanism, a metadata interrogation mechanism, a caCORE-compliant Application Programming Interface (API) and a set of shared services. These components act together to implement the principal functionality of the system. The functional components consist of a series of service objects that provide a consistent interface to web tier controllers. There are three primary functions such as "Query", "Manual Annotation" and "Import Management". The object layer of the business tier consists of services the services required for managing domain objects. The principal functions of this layer are to provide O/R mapping capabilities via caCORE SDK generated and custom code. The resulting objects present a unified model-based interface of the domain to the functional components so that they need not be concerned with the physical database implementation. In addition to the O/R mapping capabilities, the object layer also provides an API into the domain objects as required by the caBIG silver-level compliance specification. This API is generated using the caCORE SDK. Metadata interrogation capabilities can also be accessible from the object layer. The required metadata are accessible via the caDSR. However, there may be some additional metadata necessary for the rendering of user interface components that is application-specific. This tier also provides shared services for logging and audit capabilities, authentication and authorizations services via the NCI Common Security Module (CSM) and caDSR access via the NCI Clinical Infrastructure Application Framework (CIAF) module. The services will be implemented generically so as to potentially be reusable by other caBIG™ components. The data tier consists of domain database that houses the clinical annotations data, the security data (users, groups, roles, protected elements, etc.) and a staging area for import data. The database is in Oracle with a MySQL implementation.

The integration tier integrates with caTISSUE CAE in one of the following two ways:

#### Data import

**Figure 8:**
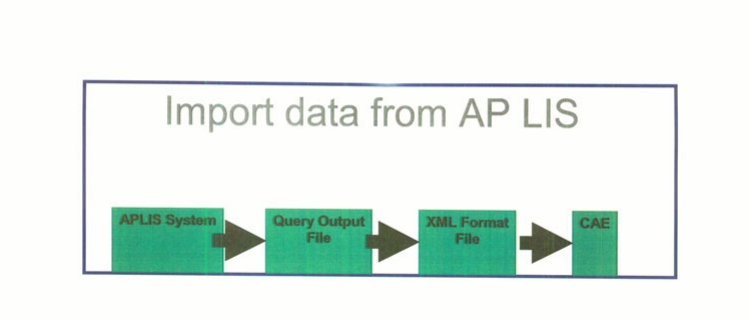
Data import from Anatomic Pathology Laboratory Information System to the caTISSUE Clinical Annotation Engine web interface.

Anatomic Pathology Lab Information Systems, Cancer Registries, caTIES and other systems that hold tissue-related data can add cases or annotation data to the system by exporting their data into a published, XML-based format. The data can then be imported into the annotations database using the web-based import management capabilities provided by the CAE system (Figure 8).

#### Application Programming Interface (API)

The CAE system also provides a caBIG™ – compliant API for accessing domain-object data, which can be utilized by caTISSUE Core and other future caBIG™-compliant systems to access and search on annotations data directly.

Currently we are developing version 2.0 of the CAE system. This new version of the tool will provide improvements along two primary fronts. First and foremost, while the 1.X versions of the tool supported only the base hierarchy of Participant->Accession->Specimen->Sub-specimen, version 2.0 will support the annotation of any network of related entities. The entity types and their relationship are specified in a UML model and generated along with the annotations. The CAE system has been adapted to handle the model that it is given. Secondly, the specification and management of metadata within the systems has been enhanced, giving researchers and bioinformaticians greater flexibility over the format of the attributes and items displayed on the user interface.

The MVB Query Tool is developed based upon an early release of CAE 2.0. The CAE team has developed this release "from the bottom up": i.e., the database and data management tiers and the API (Application Programming Interface) to these tiers are complete. The CAE 2.0 API uses the underlying data tier to provide a mechanism for performing queries on an annotated network of related entities. Additionally, the MVB query tool includes a custom built user-interface that calls upon the API to perform queries and renders the results.

**Figure 9:**
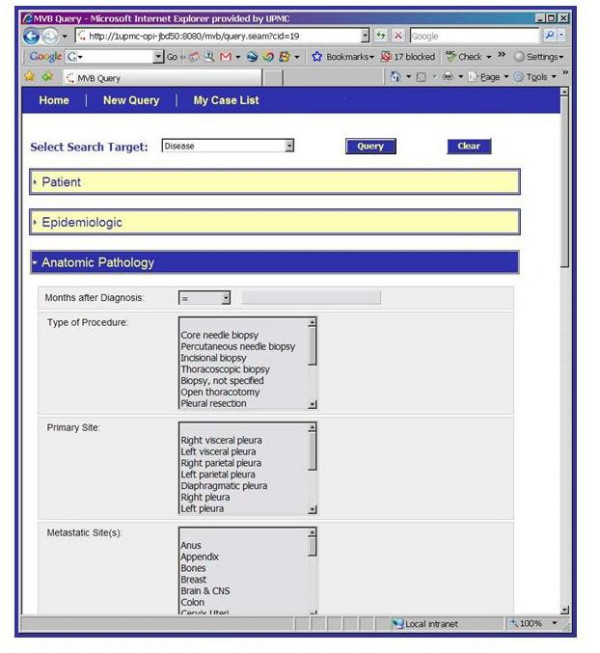
The caTISSUE Clinical Annotation Engine Query interface for Mesothelioma Virtual Bank.

**Figure 10:**
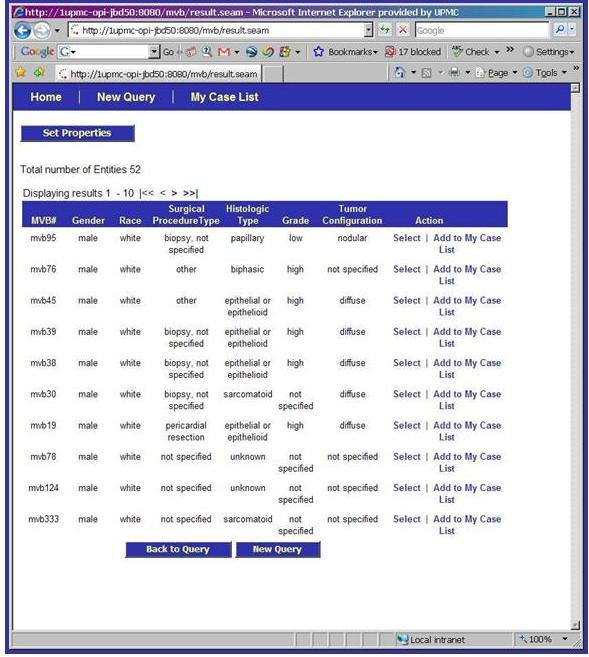
Detailed view of a queried case on the caTISSUE Clinical Annotation Engine Query result interface for Mesothelioma Virtual Bank.

The entity model that is the basis of the MVB Query Tool is illustrated in Figure 9, 10, 11. This model has replaced the strict hierarchy that is at the foundation of the previous version of CAE. The new model includes constructs for more explicitly representing disease, treatment and outcome data. The MVB Query Tool provides a user interface for querying for specimens or cases based upon attributes of or annotations attached to any of these entities.

**Figure 11:**
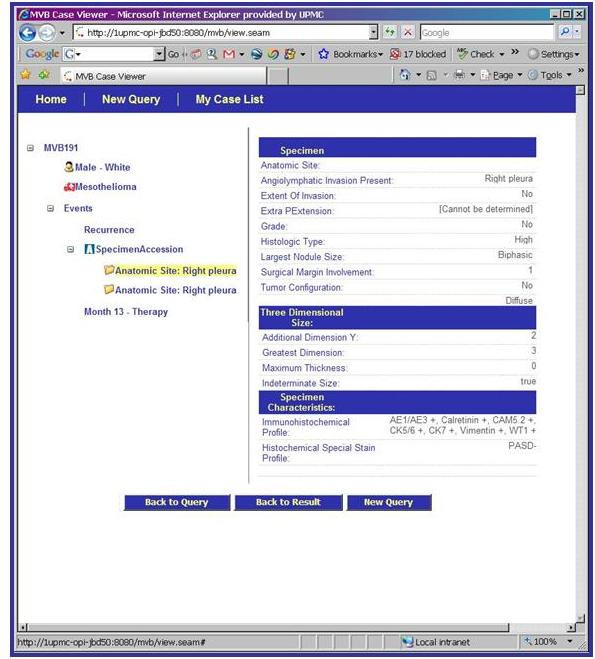
Detailed view page of a queried case on the caTISSUE Clinical Annotation Engine Query result interface for Mesothelioma Virtual Bank.

### Evaluation of CDEs by respective domain experts

The evaluation phase of the CDEs is an ongoing effort that allows for the examination of the quality of MVB data collected by the respective Domain Experts, Coordinating Committee and the members of the Research Evaluation Panel.

## Results

### Inventory of resources

Archival specimens from biopsies and resection from 1981 to present were identified from the pathology records. At the time of compilation of this manuscript, there are over 400 annotated cases of pleural, peritoneal and pericardial mesothelioma specimens that are available to the research community. The majority of these cases consist of archival paraffin blocks from surgical patients treated between 1981 and 2006, including one paraffin tissue microarray (TMA). The TMA has been designed to facilitate identification of markers differentially expressed in primary mesotheliomas and metastatic lesions. Samples from 41 patients have been incorporated in the TMA (Please refer Additional file 6 for the details of the TMA). The Resource has also accrued DNA and blood samples in 136 cases with clinical annotation. The entire workflow of Tissue Bank Workflow for MVB across the institutions is illustrated in Additional files 7 and 8.

### Development of the Common Data Elements

With guidance from the Research Evaluation Panel and the Coordinating Committee, the CDE subcommittee was primarily involved in the development of CDEs for demographics, epidemiology and clinical history. These included specimen-level annotation describing the overall case where a bio-specimen was collected, block-level annotation which records information on individual pieces or sections of the bio-specimen banked, and follow-up information about treatment, vital status, and clinical recurrence.

### Development of metadata for CDEs

Metadata is additional data developed to describe a specific CDE by following the ISO standard, which specifies that metadata should have a qualified name or identifier, an authority who registers the name, a versioning history (allowing for modifications), a language or origin, a statement relating to usage, a data typing statement, and a definition that is unambiguous. The MVB data dictionary describes each of the common data elements generated by following the ISO standard for meta-data. The most current version of the CDE data dictionary and the data collection template can be accessed at the MVB public database (Please refer to Additional file 4).

### Semantic interoperability of the system

Most tissue bank databases which contain clinical data generally lack the ability to exchange information in a common format (syntactic interoperability), the ability to understand and use the information once it is received by other systems (semantic interoperability). Another problem is the panoply of ways that similar or identical concepts or data are described by different users even within the same institution as well as across institutions. For example, a data element called "grade of tumor" can be collected using various formats (e.g. some collect "grade-1, grade-2, and grade-3" vs. others who collect "low grade, intermediate grade, and high grade"). Such inconsistency in data descriptors makes it nearly impossible to aggregate and manage even modest-sized data sets in order to perform basic queries. As a result, these systems are neither uniform nor flexible. They are incapable of performing exchange or sharing of information as unambiguous interpretation of the information is not possible without semantic and syntactic interoperability.

The MVB database is based on an informatics model that aids in developing and conveying the semantic interoperability by describing the common data elements in the form of metadata or data descriptors (about the content, quality, condition, and other characteristics of the data) and by using a controlled vocabulary and ontology in order to make the data understandable and sharable for end-users. Each common data element is associated with an object or concept, attribute, and valid value(s). For example, "patient age at diagnosis" is the CDE that is made up of "patient" (object), "age at diagnosis" (property) and the representation (value domain) in "years". Specifically for each of the approved CDEs, the data collectors need to know: 1) the fundamental definition of the data element (i.e., date of diagnosis), 2) how that data element will be collected (e.g. 11/2003 vs. Nov. 2003 vs. 11/03, etc), 3) what are the consensus acceptable values or codes are for the data element (e.g., precise date of birth, not calculated from clinical records where the "patient appears to be a well developed 75 year old"), and 4) what the acceptable data format is for inclusion into the central database (e.g., dates as integers not character strings). Although the concept of formalized metadata is fairly straightforward, it has rarely been incorporated by clinical and research groups building translational research databases [13,16].

The CDEs developed by the MVB are ISO/IEC compliant (International Standards Organization/the International Electrotechnical Commission) [32]. The MVB defines a number of fields and relationships for metadata registries including a detailed metamodel for defining and registering items, of which the primary component is a data element. By taking this approach, greater flexibility will be given to individual institutions than with the current limitations placed in data collection methods and work flow. As another example, for the CDE "Patient race", the object is "patient"; the property is "race" and the valid values are a list of character strings ("Caucasian", "African American", and "Asian", among others). If legacy databases from one institution use "African American" and another institute uses "Black" as a valid value for the "race" field and both their CDEs and metadata are properly mapped to the central database, then when researchers query for "African American" cases in the user interface, the result would display the total cases ("African American" plus "black") available from both institutions, because both terminologies are semantically integrated with the accepted system. Hence, the overall advantage of the MVB model includes the shared responsibilities of individual institutions for services and implementation of the required standards and vocabulary that would foster data sharing effortlessly.

### Re-evaluation and Quality Control of CDEs

Annual re-evaluation and quality control of the CDEs of the MVB will be conducted to determine which CDEs are most useful for routine tissue and data collection and for long-term update. If any desired CDE are found to be poorly collected from a quality control or practical standpoint, a process will be initiated to modify the data collection process through discussions with the cancer registrars and data managers. The Coordinating Committee and the Research Evaluation Panel will ensure that each participating institution will have a data manager responsible for quality control and the re-evaluation of the resource as a whole periodically to meet the needs of the mesothelioma research community. The CDE sub-committee will be charged to add new CDEs based on the needs of the researchers and addition of new resource materials (e.g., Tissue Microarrays). Additionally, the Coordinating Committee will add dates to many of the CDE categories to enable the examination of the timeline of major events that may occur for each patient during his course of disease. This "event table" has resulted in a better picture and assessment of the inter-related characteristics of patient treatments and outcomes. Once the changes to the CDEs are approved, the database will be modified accordingly. The existing data will be re-mapped and migrated to the revised CDE model.

## Discussion

Advances in molecular biology and systems biology in medicine have been the driving force that motivates development of biorepositories to provide well characterized and highly annotated tissue samples for basic science, clinical, and translational research. Clinical annotation – the association of demographic, epidemiologic, pathologic, treatment, progression, molecular and outcome datasets – is central to the success of the repositories as such annotation allows samples to be better matched to the research question at hand and experimental results to be better understood and verified [1-3]. To facilitate and standardize clinical annotation of biospecimens and to automate the process of annotation, the MVB project team identified the NAACCR standard [23], and CAP cancer protocol and ADASP guidelines for mesothelioma as the well accepted, important and standardized sets of data elements in their respective domains [24,28]. These standards consist of a series of reporting guidelines for diagnostic pathology reports and outcome related descriptors for the vast majority of human malignancies. Each CAP guideline is comprised of checklists with the data elements to describe the gross as well as microscopic attributes of the neoplasm including pathological staging and perifocal reactions such as margins, angio – as well as perineural invasion with specified valid values for each data element that are important for clinical decision-making and prognostication of individual cases.

The initial process of building a biospecimen resource with high quality specimen annotation involves considerable time and commitment from domain experts from various disciplines. Open discussions and input from all potential parties with a stake in the outcome is crucial to any such developmental work. The process of developing the CDEs for the MVB has validated that this approach can successfully lead to the implementation of robust human mesothelioma-related CDEs that guide the collection of high quality data for the research community. Clinicians and Epidemiologists (MVB CDE subcommittee) were primarily responsible for providing the foundation of data elements as they reflected the current standard of information used in patient care decisions, while attempting to project at least five years into the future for additional data that may become clinically significant. Likewise, research scientists provided input on data elements that would be crucial in the evaluation of current or proposed research related to mesothelioma with respect to the diagnosis, prognosis, and management of mesothelioma. Thus the result was the creation of datasets that should provide value to the end users, the research community, for years to come. Additionally, it was very important to include in CDE development the tissue bankers, data managers, and cancer registrars who are the main data collectors for the tissue banking resource. Their input on the types of data and data descriptors available for collection proved to be crucial in aggregating high-quality annotation data for the biospecimens. Moreover, the definitions of the CDEs and their associated descriptors need to be clearly understandable and consistent to all those who collect data. For example, to collect quality data, the curators need to understand 1) the fundamental and correct scientific definition of the data element (i.e., date of diagnosis, histopathologic type, etc), 2) the way the data will be collected (e.g. 11/2006 vs. Nov. 2006 vs. 11/06, etc), 3) the consensus acceptable values or codes for the data element (e.g., precise date of birth, not calculated from clinical records where the "patient appears to be a well developed 75 year old"), and 4) the acceptable data format for inclusion into the database (e.g., dates as "integers" not "character strings"). Through the use of ISO compliance and accepted data standards the goal of collecting annotation data of high quality was achievable. The consensus approach used by MVB was critical to successful CDE creation. Although the concept of formalized metadata is reasonably straightforward, it has been rarely integrated by clinical and research groups building databases. This study of CDE development and deployment shows that well developed CDEs can benefit comparative research and in-depth analysis of data among multiple institutions and studies. The only way in which information from multiple databases can truly be shared and made useful is through the careful use of unambiguous, clearly defined, uniform and consistent metadata. Informaticians and database developers provided the structural link that brought the CDEs together in the database, addressed technical issues, and provided guidance related to implementation of the CDEs at local institutions. Furthermore, having the ability to collect high-quality data elements that have been agreed upon by a research evaluation panel is crucial for the overall quality of quantitative analysis of data. Collecting simple, yet uniform and comprehensive data annotations in a database for research with various capabilities of collecting the data (manual review of medical charts, cancer registry systems, and interfaces to legacy systems), vastly increases the power of research efforts and has the potential to identify the natural history, common trends and issues in cancer diagnosis and management.

## Conclusion

Recently, there have been an increasing number of local, regional, national and international tissue banking efforts that have promoted formation of large research consortia and encourage these groups to share both tissue and data. Currently, many tissue banks such as the CPCTR, PCABC, caBIG, CBCTR, CHTN, CFR, SPOREs, and EDRN involve multiple institutions [4,6-8,10-13,15,19-21]. These biorepositories vary in their data collection and tissue collection methodologies and involve common malignancies. However, the necessity for well-annotated tissues that can be re-annotated with experimental data has driven many of these multi-institutional collaborations to develop standards of sharing data with other groups. In addition, so far as human mesothelioma is concerned, there is no such biorepository available with CDEs based upon clearly defined, uniform and consistent metadata. Currently, the MVB CDEs are specifically related to the available mesothelioma tissue resources with multimodal datasets. Publications resulting from the use of MVB tissues will allow for the results to be correlated to or compared with other studies using similar CDE standards. Other initiatives such as the CPCTR, EDRN, PCABC, and caBIG can perform follow-up studies by linking their results to MVB-derived studies by using the common MVB CDEs. This also allows for meta-analysis of data in a more efficient and logical way across studies through the MVB CDEs, resulting in improved statistical power and further detailed analysis. Thus, expanding the MVB dataset by combining tissue with molecular, genotype, gene expression, and image associated data will have remarkable impact on cancer research community and will help in understanding the genesis, evolution and progression of disease through molecular validation and high-throughput genomic and proteomic analysis, which will eventually reduce the burden and suffering of cancer.

Based on the experience of developing CDEs for the MVB, the following sequential strategies can be recommended for other research groups involved in future CDE development efforts.

• Decide which CDEs the resource will need using a committee-driven consensus process that includes all major stakeholders.

• Utilize as a starting point similar CDE initiatives already developed by others and build upon their standards.

• Consult a variety of experts, including those that will be directly collecting the data – particularly tissue bankers, research nurse coordinators, cancer registrars and data managers.

• Draft a CDE data dictionary which includes structured data with precise data field definitions, attributes and valid values.

• Modify or approve CDEs after discussions with all key personnel and end users and build consensus among them and any other external experts.

• Create corresponding data entry paper forms/data entry interface to a central database.

• Implement CDEs.

• Test/Pilot phase: sharing of data with central database.

• Every year after initialization set accrual target and re-evaluate and perform quality control measures on CDEs and their values.

• Establish and continually develop quality assurance and quality improvement protocols to fully develop the CDEs.

## Competing interests

The author(s) declare that they have no competing interests.

## Authors' contributions

MJB, SKM, ATM, WA and GF contributed equally to the first draft of this manuscript. MJB, the chair for the Coordinating Committee, is responsible for leading the efforts of developing the requirements for the central database. MJB, SKM, ATM, WA, GF, ET, AAP, RD, JM and HIP have contributed in study design, implementation and quality assurance of the database and tool. LS and AKP have contributed in the development and implementation of software tools for the data annotation and query engine in the web-based interface, and incorporation of other existing standards. ET, SBW and GF have played an important role in implementation of Cancer registry data standards into the database and collection and quality assurance of follow-up and epidemiological data. NBW is the overall Project coordinator for this project. PK and EM are the lead for the marketing sub-committee for this project. All authors have reviewed and commented on successive drafts of the manuscript and have provided the first author with approval of the final manuscript.

## Pre-publication history

The pre-publication history for this paper can be accessed here:



## Supplementary Material

Additional file 1 IRB protocol for the Mesothelioma Virtual Bank project. Click here for file

Additional file 2 IRB approval letter for the Mesothelioma Virtual Bank project. Click here for file

Additional file 3 Honest Broker services for Tissue Banks and Clinical data. Click here for file

Additional file 4 Common Data Elements data dictionary with metadata and valid values. Click here for file

Additional file 5 Data collection template for the Mesothelioma Virtual Bank project. Click here for file

Additional file 6 Paraffin Tissue MicroArray template for the Mesothelioma Virtual Bank project. Click here for file

Additional file 7 Tissue Bank Workflow for MVB across the institutions. Click here for file

Additional file 8 CDC MVB Tissue Request Work Flow Chart. Click here for file
