# Supplementary material for: The development and deployment of Common Data Elements for tissue banks for translational research in cancer – An emerging standard based approach for the Mesothelioma Virtual Tissue Bank
Source: BMC Cancer. 2008 Apr 8;8:91. doi: 10.1186/1471-2407-8-91 (PMC2329649; doi:10.1186/1471-2407-8-91)
Supplement: Additional file 1 — IRB protocol for the Mesothelioma Virtual Bank project. [file 1471-2407-8-91-S1.pdf]

Mesothelioma Virtual Bank for Translational Research

Objective and Specific Aims:

The objective of the Mesothelioma Virtual Bank (MVB) for Translational Research is to maximize the effectiveness of data and biospecimen collection for mesothelioma. MVB will serve as a resource that will allow researchers real time access to clinical data associated with tissue specimens from the registry, thus expanding scientific discovery and effective treatments to benefit the mesothelioma research and patient community. The specific aims of the MVB are to:

(1) **Develop and maintain a virtual mesothelioma patient registry and tissue bank.** We will collect patients' clinical and demographic data, including data determined to be necessary for diagnosis and inclusion in the registry and tissue bank; and biological samples including (but not limited to) biopsy material (fixed or frozen), serum white blood cells and nucleic acids. We will establish standards for participants to post clinical data, demographic data, and available samples in a distributed network allowing databases of individual participating institutions to be accessed together, forming a single virtual database.

(2) **Establish the mesothelioma patient registry and tissue bank as a resource for the clinical science community.** Our MVB cooperative group will establish a process for qualified applicants to access information in the database and to request available samples for use in legitimate research. Together, with National Institute for Occupational Safety and Health/Center for Disease Control (NIOSH/CDC), the MVB consortium working in collaboration with TRAC (Translating Research Across Communities) will publicize the availability of this resource to prospective users; not only with a sound marketing plan but through extensive interaction with advocacy groups. This will allow us to effectively communicate effectively about the resource to the scientific community, and to the patients whose lives are affected by this deadly disease. Moreover, the MVB cooperative group in collaboration with TRAC will seek additional resources in the form of collaborations, services, and funding to further the goals of the Mesothelioma Community by creating a model national resource for Translational Research.

(3) **Document the usefulness of the mesothelioma virtual registry and tissue bank to the scientific community in the conduct of studies that address the etiology, mechanisms, diagnosis and treatment of malignant mesothelioma.** The MVB cooperative group will maintain a record of requests for access to the database; requests for use of tissue samples; tissue samples distributed; scientific abstracts, presentations, and publications resulting from use of the registry and tissue bank; and patents or products resulting from use of the registry and tissue bank.

Background and Significance:

One of the clear barriers to research of the type proposed in this IRB is that individual institutions do not have the necessary numbers of specimens and carefully collected clinical outcomes to perform larger scale investigations and clinical trials. The purpose of this MVB is to facilitate collaborative research among the seven member institutions as well as provide access to other institutions both within and outside of the consortium eventually.

As part of the rationale that the NIOSH/CDC has asked us to pursue this effort is to maximize the effectiveness of data collection and serve as a resource that will allow researchers real time access to clinical data associated with tissue specimens from the registry, thus expanding scientific discovery and effective treatments to benefit the mesothelioma research and patient community. Access to these highly annotated clinical tissue specimens from seven leading cancer research centers in Pennsylvania acting as a single unit will make considerable strides to facilitate this activity.

Malignant mesothelioma is a rare form of cancer that presents as a malignant growth in the sac lining of the chest (the pleura), or the abdominal cavity (the peritoneum) or the lining around the heart (the pericardium). Asbestos exposure through inhalation of asbestos fibers is the main cause of mesothelioma. For many years, American workers have been exposed to asbestos in the workplace, including workers in industrial and building trades and Navy personnel. Although the use of asbestos has been significantly reduced since the 1970s, mesothelioma is still a significant occupational health burden. Each year, in the United States, 2,500 to 4,000 people are diagnosed with mesothelioma. This is because the latency period for mesothelioma is 20-40 years, with the average patient age of 50 years at the time of diagnosis. Furthermore, the risk of asbestos exposure still continues today in many occupational settings, and in buildings such as homes, offices and schools, in which asbestos was previously used or installed and remains present. Thus, experts predict mesothelioma incidence in the U.S. will continue to increase for another 10-20 years. To date, there is no cure for mesothelioma and effective treatments are limited. Increased research efforts on mesothelioma will aid in the discovery of more effective treatments to combat this deadly disease.

There have been many attempts to provide better infrastructure for cancer tissue acquisition and research distribution. Unfortunately, despite the best of initial intentions by many banking sites the actual number of publicly accessible cancer tissue resources is scarce. Of the selected public resources today, our team has been involved with two groups, the Cooperative Prostate Cancer Tissue Resource **(IRB# 020527)** and the Pennsylvania Cancer Alliance Bioinformatics Consortium **(IRB#0303119)**, that provides the level of clinical information needed to support outcomes based investigations. The lack of tissue available for research and access to de-identified clinical data is due to multiple factors including:

- no incentive to share tissue outside of region where they are collected,
- scarcity of tissues due to lack of cooperation from surgery, pathology, oncology and clinical/translational/basic science researchers who may compete for the same resources,
- scarcity of resources and a general lack of appreciation for the costs involved in well developed banking infrastructures,
- difficulty of obtaining appropriate control tissues,
- extremely limited access to tissues from patients with advanced (metastatic) cancer,
- misconceptions of regulatory and proprietary rules governing tissue banking; specifically IRB, consent, honest broker, de-identification and ownership issues
- inadequacy of the informatics infrastructure to support tissue banks and virtual networks of collaborators with banking collections

The MVB team, led by the University of Pittsburgh, has systematically tackled these issues and the methods we have utilized are described in the preliminary results section of this proposal. In general, however, our experience has taught us that the tissue banking team must create an environment that fosters collaboration between surgeons, pathologists, oncologists, researchers and patient focused advocacy groups. We believe strongly that biospecimen resources and

their clinical annotations are among some of the most powerful resources fueling translational research. The MVB proposes to implement a virtual registry and tissue bank system for the nation focused on advancing our understanding of the tumor biology of mesothelioma with the goal of facilitating the discovery of preventative interventions, new treatments and ultimately cures for this uniformly lethal disease. The National Institute of Health (NIH) Roadmap stresses the importance of collaborative networks of researchers and the sharing of biospecimen resources and the National Mesothelioma Virtual Patient Registry and Tissue Bank program of NIOSH/CDC clearly aims to facilitate that vision.

Research Design and Methods:

This proposal is a cooperative mechanism and will have a governing MVB Coordinating Committee (MVBCC), composed of Drs. Becich, Dhir and Taioli from UPCI; a patient advocate, one member from each of the accruing sites and two members from the CDC/NIOSH including the Program Coordinator. The role of the MVBCC will be to oversee and guide MVB's development and ongoing activities.

The system is designed as a virtual bio-repository that gathers information on banked tissues and patients in research trials – including clinical and outcomes data – from members of the MVB. The MVB will be centrally coordinated by the University of Pittsburgh in collaboration with the MVB Coordinating Committee. The MVB consortium has already enrolled eight institutions including: Abramson Cancer Center of the University of Pennsylvania, Drexel University, Fox Chase Cancer Center, Geisinger Health System, Kimmel Cancer Center of Thomas Jefferson University, Penn State Cancer Center, Windber Research Institute and the University of Pittsburgh Cancer Institute (UPCI). Each member will also be responsible for getting proper IRB approval at their respective institutions. At which time, this data will be de-identified at the local institutions and made available in a central data warehouse for visualization and query.

In addition, participating institutions will identify and enroll mesothelioma patients and record them in the MVB database. They will collect patients' clinical and demographic data, including data determined to be necessary for diagnosis and inclusion in the registry and tissue bank; and biological samples including (but not limited to) biopsy material (fixed or frozen), serum white blood cells and nucleic acids. The letters of support provided by the participating institutions prove we have a track record of working together and can quickly accrue these specimens for sharing with the mesothelioma research community. The participating sites already have in place:

1. Familiarity with Common Data Elements to support MVB.
2. Standard operating procedures for the collection, storage and handling of biospecimens.
3. Standardized IRB and "universal" consent forms for institutional regulatory and compliance issues.
4. Honest Broker Models to allow processing, deidentification and data sharing between coordinating sites, collection sites and the research community.
5. Material transfer agreements and intellectual property agreements.
6. Established standard operating protocols for the IATA regulated distribution and shipping of biospecimens.

The Following Honest Broker System or Process will be utilized: Department of Biomedical Informatics and Center for Pathology Quality and Healthcare Research: Health Sciences Tissue Bank (HSTB), Clinical Outcomes, and Cancer

Registry (UPMC/IRB Honest Broker Approval Number: HB015).

The workflow for entering data into the virtual bio-repository will be as follows:

1. The local (physical) tissue bank will identify cases appropriate for inclusion in the MVB's virtual bio-repository (warehouse).
2. The local (physical) tissue bank will pre-process data on these cases. The most important component of pre-processing will be de-identification. All de-identification will occur at the local banks. No identifiable data will be sent to the virtual bio-repository (warehouse).
3. De-identified data will be entered into the warehouse through a web site. The data entry web site uses radio buttons, combo boxes and other highly constrained data elements.
4. The local (physical) banks will label each case with a de-identified number. This number will be used to link the information in the warehouse to the cases in the local banks. The linkage codes will be stored locally, using appropriate electronic and physical safety measures. Only the local banks will have access to these linkage codes.
5. The warehouse will contain very minimal demographic data and will comply with all HIPAA requirements. In particular there will be no:
   - No Patient names
   - No Medical Provider names
   - No Patient Addresses (including Zip Codes, States and Countries)
   - No social security numbers or any other personal Ids.
   - No accession numbers or other medical Ids.
   - No dates (including no date of birth or date of diagnosis).
   - All time periods will be entered as plus or minus months from diagnosis. For example the "date of first recurrence" will not be entered as a date but rather as "months from diagnosis" (i.e. Date of First Recurrence = 67). In local tissue banks will not enter any fixed dates into the virtual bio-repository (warehouse).
   - Currently the system does ask for age at diagnosis. There is a discussion about constraining this by decade (20s, 30, 40s, etc).
   - There will be no free text of comment fields. All data will be entered into highly constrained "synoptic" fields.
   - In summary, no identifiers

6. Access to the data entry application is controlled by user name and password.
7. Cases entered into the virtual bio-repository will be scanned for logical errors (e.g.: first recurrence before diagnosis etc.).
8. The workflow for querying the warehouse will be as follows:

Initially, access will be limited to members of the MVB using a user and password system. The data in the warehouse will eventually be made available to the public using the model of the NCI's Collaborative Prostate Cancer Tissue Resource and the Pennsylvania Cancer Alliance Bioinformatics Consortium. A single individual at each facility will be able to provide user names and passwords for researchers at that institution. Significantly, access to the data will be through highly constrained "click and point" interface. There will be no mechanism for ad hoc query. The output of the

system will therefore be highly constrained. Should a researcher find tissue samples that may be useful in ongoing or proposed research, the researcher will have to contact the MVB and the local bank(s) that hold the tissue. Tissue will not be released until after:

- IRB approval at the researcher's institution – for patient safety
- IRB approval at the tissue bank institution – for patient safely
- Approval of the MVB Scientific Review Committee – to determine if the proposed research has validity and justifies the use of potentially valuable tissue resources.
- Approval of the local (physical) tissue bank - As "owner" and guardian of the tissue specimen (the local banks may have their own review committees)

In summary, the Mesothelioma Virtual Tissue Bank project is seeking to create a central resource through which researchers can find quality annotated biospecimens. The resource will have no access at any time to patient identified data and tissue will not be made available to researchers without IRB, Scientific Review Committee approval and local control.

Human Subjects:

In addition to being an information- sharing project, we will now store tissues in repositories located at each institution. We will prospectively consent using our universal tissue banking consent form (see Appendix C: "Banking and Disbursement of Tissue and other biologic materials" **(IRB number 0506140)** which will allow us access to serum and tissue specimens any prior or future specimens on the patient's consented with mesothelioma at all eight institutions. Individuals to be approached for participation in the MVB Registry will include all adult (age $\geq$ 18 years old) patients who are receiving or seeking medical care for mesothelioma. Note that this study will not include children. All individuals approached for participation in the MVB Registry shall be able to read and comprehend English. Due to the complexity of state and federal requirements governing the participation of prisoners in research, prisoners-patients shall not be approached for participation in the MVB Registry. Since participation in the MVB Registry does not involve a risk of physical harm, women of childbearing potential will not be queried as to pregnancy status or tested for pregnancy. There are no additional inclusion/exclusion criteria.

The racial, gender and ethnic characteristics of the individuals approached for participation in this MVB Registry shall reflect the demographics of patients receiving or seeking medical care at the mesothelioma clinics at member institutes of this consortium. We shall attempt to recruit participants in accordance with these demographics. No individuals shall be excluded from participation in the MVB Registry based on race, ethnicity, gender or HIV status.

a. **Inclusion of Children**
   This study will not include children.

b. **Recruitment procedures**
   Biological materials collected for this project will be prospectively consented as

mentioned above using the IRB approved protocol #0506140.

c. <u>Risk/ Benefit Ratio</u>

<u>There will be no risks for the patient.</u> This study will not require anything in addition to what is already being done as standard of care.

The study will help improve the overall understanding of prostate cancer, breast cancer, and melanoma. **It will not provide any direct benefit to the patient.**

Recruitment Procedures:

All patients who are receiving or seeking medical care for mesothelioma at UPMC/University of Pittsburgh facilities will be invited to have a sample of their tissue or biological specimens included in the Health Sciences Tissue Bank. The HSTB procures excess materials for research purposes. This aggregation is performed after adequate materials have been obtained for diagnostic purposes. These research tissues are provided to investigators only after clinical diagnostic issues have been accomplished (report signed out on that specimen).

This research material procured is from two different sets of tissue samples:

I. Retrospective collections:

1. The retrieval of samples from archived residual biospecimens that have been removed for clinical treatment and pathology diagnostics as well as retrospective review of associated medical records for annotation purposes fall within the IRB's exempt category. The MVB registry will utilize the key members of the HSTB and the certified honest broker system for identifying and enrolling eligible cases into the MVB database. The HSTB is IRB approved for collecting excess tissue and biological materials (IRB approval 981252). In addition, the HSTB is a certified honest broker facility (HB 015). This honest broker certification was part of a larger, more comprehensive, submission, incorporating the HSTB, the Cancer Registry, the oncology groups (medical oncology and radiation oncology), the Outcomes group, and Pathology and Oncology informatics. This honest broker entity essentially covers all aspects of biological specimen and data needs for MVB researchers. The combined capabilities of the HSTB and this honest broker facility should be able to address almost all needs of researchers, in a manner compliant with current legal and ethical considerations, consistent with current OHRP recommendations.
2. Majority of the tissues in the pathology archives will be paraffin blocks. Each case may have multiple blocks associated with various histology (tumor vs. non-tumor areas) that maybe of interest to potential researchers. Since the number of cases/blocks available maybe limited for mesothelioma, the MVB group will also create tissue microarrays (TMA) in order to get maximum the utilization of the limed resources for the research community. Certified honest brokers from the group will identify and retrieve potential cases used for constructing the TMAs by the HSTB's histotechnologists.

II. Prospective collections:

1. **Consented patients**: These patients are approached for tissue donation for research purposes in their doctor's

offices. Potential participants will be approached by a member of the Clinic staff and will be asked to review a copy of the informed consent form prior to being seen by a Clinic physician (i.e., MVB Registry investigator). The universal consent form used by the Health Science Tissue Bank (Consent Form titled "Banking of Tissue and Biological specimens for Research use by the Health Sciences Tissue Bank" see Appendix #0506140) will be used for consenting and enrolling potential prospective MVB participants.The Clinic physicians (i.e., MVB Registry investigators) will review the informed consent form with potential participants and address any questions or concerns prior to obtaining written informed consent for their tissue/biospecimens donation to the HSTB. The Clinic physicians (i.e., MVB Registry investigators) will also address any future questions or concerns of MVB Registry participants. In addition, *the potential participant will be informed that it is likely that the research studies in which their tissue/biological specimen will be used will be directed at* mesothelioma research. *However, it is possible that their tissue/biological sample may be used in research studies directed at other diseases or conditions* (as stated in the HSTB consent form). The specimens from consented patients are stored using a linkage code that allows subsequent additional data aggregation. We also utilize a bar coding system. This further addresses issues pertaining to patient confidentiality and identification of specimens.

2. **Excess tissue specimens from patients not specifically consented for research tissue aggregation:** These patients have not been approached for consent. The excess tissue specimens are initially collected and stored for potential clinical purposes, in case additional tissue sections might need to be evaluated for diagnostic purposes. These specimens are moved to the HSTB research repository once the clinical sign-out is accomplished. Since these patients have not been consented, these specimens are annotated only with the surgical pathology report. The annotating surgical pathology report contains de-identified (HIPAA compliant) data. This annotating report does not contain the surgical pathology number or any other mechanism to identify the patient. No additional data aggregation is permissible for these specimens.

3. **Mechanism for dealing with refused consent:** Rarely a patient refuses when approached for consent for research tissue and biological specimen aggregation. The doctor's offices communicate this back to the HSTB. No residual biological material is collected for research purposes from these patients. There might be instances where information regarding refusal of consent is not communicated from the doctor's offices to the HSTB. This is recognized as a potential problem. However research samples that do not have a consent form are anonymized. The identity of the patient would therefore be removed from the sample.

Only the medical record information of patients who have provided directly their written informed consent for banking tissue and biological specimens for research use by the Health Science Tissue Bank will be placed in the MVB Registry. For those cases that are eligible to be enrolled in the MVB Registry, there will be a mandatory data field regarding consenting that would be appropriately filled within the MVB Registry Database data entry process. The participation of patients who are mentally incapacitated (e.g., comatose, unresponsive) will not be sought (i.e., during the period in which they are mentally incapacitated).

Potential Risks of Research Registry Participation:

There are no risks of physical harm associated with participation in the MVB Registry. Participation in the MVB

Registry does involve the potential risks of a breach of confidentiality of the medical record information and associated privacy of the participants. Such risks will be minimized by 1) removing direct participant identifiers (i.e., names, social security numbers, medical record numbers) from information stored in the MVB Registry database; 2) securing, in a separate location, and limiting access to information linking codes (i.e., linkage codes) assigned to the Registry information with direct participant identifiers; and 3) limiting access to information contained within the MVB Registry to investigators who are affiliated at member institutions of the MVB consortium study.

The data and safety monitoring plan for the MVB Registry will involve routine (i.e., quarterly) monitoring by the Principal Investigator of 1) the removal of direct identifiers from information contained with the MVB Registry; 2) the documentation of investigator access to the MVB Registry; 3) the security of the database linking the MVB Registry linkage codes with participant identifiers and the documentation of investigator access to this database; and 4) any conditions that may negatively impact the confidentiality of information contained within the MVB Registry. Each member of the study team will meet with the PI and review confidentiality issues and complete a confidentiality agreement, prior to having contact with research subjects. Investigators and study personnel will meet monthly to discuss the study (e.g., study goals and modifications of those goals; subject recruitment and retention; progress in data coding and analysis; documentation, identification of adverse events or research subject complaints; violations of confidentiality) and address any issues or concerns at that time. Minutes will be kept for these meetings and will be maintained in the study regulatory binder.

As specified previously, the Principal Investigator must provide approval for a mesothelioma investigator to access the MVB registry for retrospective research studies involving the use of Registry information. The Principal Investigator must also prior approve any access of mesothelioma investigators to the database linking the Registry information to participant direct identifiers. Access for investigators to the MVB Registry for the purpose of identifying potential subjects for participation in a research study shall be granted only upon the provision of documentation that the IRB has approved the respective research study. At the time of annual renewal, a list of studies conducted using the registry will be submitted to the IRB. In addition, any unauthorized access to medical record information contained within the research registry or to the database linking the Registry information to participant direct identifiers shall be reported to the IRB.

Potential Benefits of Research Registry Participation:

There are no direct benefits associated with participation in the MVB Registry. The use of information contained within the MVB Registry for retrospective research analyses may be of future benefit to patients with mesothelioma. Participants in the MVB Registry will be informed of future research studies involving mesothelioma clinical trails for which they may be eligible. Participation in a future research study involving mesothelioma, for which separate informed consent will be obtained, may offer direct benefit to the MVB Registry participants.

Costs and Payments:

All costs associated with the implementation and maintenance of the MVB Registry shall be supported by the MVB study. No costs will be incurred by Registry participants or their health care providers. Patients enrolled in the MVB

will not be remunerated for their participation in the MVB Registry.

Investigators Qualifications:

Michael J. Becich, MD, PhD

POSITION TITLE

Chairman, Department of Biomedical Informatics
(http://www.dbmi.pitt.edu/)
Professor of Biomedical Informatics, Pathology, Information Sciences and Telecommunications
UPMC Cancer Pavilion, Rm. 305
5230 Centre Avenue
Pittsburgh, PA 15232
Office - 412-623-3941
FAX - 412-623-2814
E-mail = becich@pitt.edu

INSTITUTION AND LOCATION	DEGREE	YEAR(s)	FIELD OF STUDY
Northwestern University, Evanston, IL	B.A.	1977	Biology
University of Nebraska, Omaha, NE	Fellow	1983	Pathology
Northwestern University, Chicago, IL	M.D.,	1984	Medicine/Exp. Pathology
Washington University School of Medicine	Intern	1985	Anatomic Pathology
Washington University, St. Louis, MO	Resident	1988	Anatomic Pathology
Washington University School of Medicine	Fellow	1989	Anatomic Pathology

RESEARCH AND PROFESSIONAL EXPERIENCE

1989-1991 Instructor, Washington University School of Medicine
1991-1996 Assistant Professor, University of Pittsburgh School of Medicine, Division of Cellular/Molecular Pathology
1996-2002 Associate Professor, University of Pittsburgh School of Medicine, Divisions of Cellular and Molecular Pathology and School of Information Sciences and Telecommunications
7/99-present Director, Center for Pathology Informatics, University of Pittsburgh School of Medicine
3/00-8/00 Interim Chief of Pathology, UPMC Shadyside
3/00-12/03 Chairman of Pathology, UPMC Shadyside, Department of Pathology
11/01-present Director, Benedum Oncology Informatics Center, University of Pittsburgh Cancer Institute
9/02-present Professor, University of Pittsburgh School of Medicine, Divisions of Cellular and Molecular Pathology and School of Information Sciences and Telecommunications
01/04-present Vice-Chairman of Pathology, University of Pittsburgh School of Medicine
01/06-present Associate Director for Cancer Informatics, University of Pittsburgh Cancer Institute

Dhir, Rajiv MD
Associate Professor
Pathology, UPMC
5232 Centre Ave
Pittsburgh, PA
Phone: 412-623-1321
E-Mail:dhirr@upmc.edu

INSTITUTION AND LOCATION	DEGREE	YEAR(s)	FIELD OF STUDY
All-India Institute of Medical Sciences, New Delhi, India	MBBS	1984	Pathology
All-India Institute of Medical Sciences, New Delhi, India	M.D.	1989	Pathology

Professional Experience:

11/97-12/99 Research Assistant Professor, Pathology, University of Pittsburgh, Pittsburgh, PA
11/97-present Director, Health Sciences Tissue Bank, University of Pittsburgh Health Systems.
01/00-03/04 Assistant Professor, Pathology, University of Pittsburgh, Pittsburgh, PA
04/00-present Member, NCI coordination committee, Pathology, IRB and Marketing Subcommittees, Cooperative Prostate Cancer Tissue Resource.
7/00-present Project Leader, Tissue Resource Core for the Director's Challenge U01 funded for the "Molecular Reclassification of Prostate Cancer".
12/00-present Project Leader, Tissue Resource Core for program project on Collaborative Urologic Research in Spinal Cord Injury.
06/01-present Project Leader, Tissue Resource Core for the Lung SPORE.

07/02-present Division Director, Genito-Urinary Pathology, University of Pittsburgh Health Systems.
09/02-present Chair, Marketing committee, Co-operative Prostate Cancer Tissue Resource.
11/02-present Member, NIDDK coordination committee, Pathology and Biomarker Subcommittees, MTOPS consortium.
1/04-present Medical Director of the Tissue and Research Pathology Services Shared facility for the University of Pittsburgh Cancer Center.
04/04-present As sociate Professor, Pathology, University of Pittsburgh, Pittsburgh, PA

Honors and Awards:

1. The Smith Kline-Beecham Travel Award from the Society for Basic Urologic Research for the poster titled "An Immunohistochemical detection of standard and variant forms of CD44 expression in metastatic and organ confined prostatic Adenocarcinoma".
2. Abstract accepted for the Pathology Resident Awards competition at the fall meeting of the ASCP in New Orleans in Oct. 1995.
3. The Dr. Satya Monga award for the best paper on immunocytochemistry at the annual meeting of the Indian Academy of Cytopathologists in February 1989.
4. The National Talent Search Scheme Award, given by the National Council for Educational Research and Training, New Delhi, India.
5. The National Award, given by the Govt. of India in 1978, for performance in grade XII.

The National Award, given by the Govt. of India, in 1975 for performance in grade X.

Emanuela Taioli MD, Ph.D.
POSITION TITLE
Professor
UPMC
5150 Centre Ave
Pittsburgh, PA 15232
Phone: 412.623.2217
E-mail: taiolien@upmc.edu

INSTITUTION AND LOCATION	DEGREE	YEAR(s)	FIELD OF STUDY
University of Milan - School of. Medicine	MD	1981	Medicine
Columbia University School of Public Health	MS	1990	Epidemiology
Columbia University School of Public Health	PhD	1999	Epidemiology

Positions and Employment
2005/present Professor, Department of Epidemiology, UPSPH, Pittsburgh
Director, Cancer Prevention and Population Science, UPCI

1994-2005	Director, Genetic and Molecular Epidemiology Unit, Fondazione Policlinico Milan
1994-present	Adjunct Associate Professor, NYU Medical Center, Department Environmental Medicine
1992-94	Assistant Professor, NYU Medical Center, Department Environmental Medicine
1991-92	Epidemiologist, American Health Foundation, Department of Epidemiology, New York
1986-89	Research Fellow in Cardiovascular Epidemiology, Ospedale Maggiore - Milan, Italy
1984-86	Research Fellow in Clinical Pharmacology, Mario Negri Institute Milan, Italy
1981-84	Research Fellow in Internal Medicine, University of Milan

Appendix C

Institutional Review Board

Approval Date: June 26, 2006
Renewal Date: June 25, 2007
University of Pittsburgh

IRB# 0506140

CONSENT TO HAVE A TISSUE SAMPLE OR BIOLOGICAL SPECIMEN INCLUDED IN THE HEALTH SCIENCES TISSUE BANK

TITLE: Banking of Tissue and Biological specimens for Research use by the Health Sciences Tissue Bank

PRINCIPAL INVESTIGATOR: Rajiv Dhir, MD, Associate Professor of Pathology, (412) 623-1321

SOURCES OF SUPPORT: National Institutes of Health/ National Cancer Institute

DESCRIPTION: Because you are or will be undergoing a surgical or other procedure involving the removal or collection of tissue or biological specimens (e.g., blood, urine, saliva, etc.) for your medical care, you are being asked to give your consent to include a sample of your tissue/biological specimen in the Health Sciences Tissue Bank (HSTB). **If you consent to this request, all testing of your tissue/biological specimen required for your medical care will be completed prior to obtaining the sample for inclusion in the HSTB.**

Samples of tissue/biological specimens placed in the HSTB will be used for research studies. The specific nature of the research studies in which your tissue/biological specimen may be used will vary and are not fully known at this time. If the tissue/biological specimen is being surgically removed or collected because you have a specific disease or condition, it is likely that the research studies in which your tissue/biological specimen will be used will be directed at the same disease or condition. However, it is possible that your tissue/biological sample may be used in research studies directed at other diseases or conditions. **Your tissue is released for research studies only after careful review of the research proposal by oversight committees (e.g. tissue utilization committee/ IRB, as appropriate for the research project).**

Your sample will be stored in the HSTB in such a manner whereby it will be possible for the individuals responsible for the HSTB to connect your identity with your sample. Also, in order to use this sample in an effective manner for research, it is also often necessary that your medical information is available for review. Hence, if you agree to include a sample of your tissue/biological specimen in the HSTB, you also agree to allow individuals responsible for the HSTB to review and collect identifiable information from your medical records. **However, when your tissue/biological**

specimen and medical information are made available for actual use in research studies, it will be provided to the researchers in such a manner whereby it will not be possible for them to connect your identity with the sample or medical information.

Because it will not be possible to connect your identity with your tissue/biological specimen when the sample is being used for research, it will also not be possible to inform you of the results of such research.

If you agree to give a sample of your tissue/biological specimen to the HSTB, it will become the property of the University of Pittsburgh and its use will be under the control of the individuals responsible for the HSTB, who are listed on the first page of this consent form. Your medical information and sample will be stored in the HSTB until such time that the sample is used up or no longer felt to be appropriate for use in research studies. Your decision to provide these specimens and identifiable medical information to the HSTB, or to later withdraw from it, will not affect your current or future medical care at UPMC.
RISKS AND BENEFITS: You will receive no direct benefit by agreeing to include a sample of your tissue/biological specimen in the HSTB. However, the availability of such samples for research use is important to the future development of new treatments. There are no additional risks associated with participation. **No additional amount of your tissue/biological specimen will be removed surgically or collected for the purpose of including a sample in the HSTB.** Rather, the sample of your tissue/biological specimen that we are requesting for inclusion in the HSTB is that which would normally be thrown away after the testing for your medical care is completed. Hence, there are no additional risks if you agree to allow a sample of your tissue/biological specimen to be included in the HSTB.
COSTS and PAYMENTS: There will be no additional costs to you or your insurance company if you agree to include a sample of your tissue/biological specimen in the HSTB. You will not be paid for the inclusion of your sample in the HSTB. Use of your tissue/biological specimen for research may lead, in the future, to new inventions or products. If researchers are able to develop new products from the use of the sample that you donate to the HSTB, **you will not receive any money for this donation.**

CONFIDENTIALITY: To protect your privacy, your name will be removed from the sample of your tissue/biological specimen and medical information while it is stored in the HSTB. Your sample and information will be stored in the HSTB using a code number, and only the individuals responsible for the HSTB will be able to connect this code number with your identity. The information linking this code number with your identity will be kept by these individuals in a secure manner.

As stated above, when your tissue/biological specimen and medical information are made available for actual use in research studies, it will be provided to the researchers in such a manner whereby it will not be possible for them to connect your identity with the sample or medical information. Therefore, your identity will not appear in any articles describing the results of research studies that involved the use of your sample.

RIGHT TO WITHDRAW: You may refuse to allow us to include a sample of your tissue/biological specimen in the HSTB. Such a decision will not affect the current or future care that you receive at this institution or any other benefits for which you might qualify.

If you agree to include a sample of your tissue/biological specimen and medical information in the HSTB, you may

withdraw your permission at any time through a written request. Depending on your wishes, we can either destroy any remaining amount of the sample or continue to store your sample in the HSTB, but in a totally anonymous manner (i.e., no one, including the individuals responsible for the HSTB, will be able to connect your name with your sample). Your medical information will also be removed from the HSTB.

It is not possible for us to guarantee that we will be able to destroy any of your samples that may have been previously provided for research use since your identity will not be connected with these samples.

****

VOLUNTARY CONSENT: The above information has been explained to me and all of my questions have been answered. I understand that any future questions I have about the donation of a sample of my tissue/biological specimen to the HSTB will be answered by a qualified individual or by one of the individuals responsible for the HSTB, who are listed on the first page of this form. I also understand that I may always request that my questions be answered by one of the individuals responsible for the HSTB. The Human Research Subject Advocate of the Institutional Review Board, University of Pittsburgh (1-866-212-2668), will answer any questions that I may have about my rights as a research subject.

By signing this form I agree to allow a sample of my tissue/biological specimen to be included in the Tissue Bank for use in research studies directed at any disease or condition and I agree to allow the use and disclosure of my medical record information, as described above.

____________________________________ ____________________
Patient/Subject Signature Date

I certify that the nature and purpose, the potential benefits and possible risks associated with the donation of tissue samples/biological specimens to the Tissue Bank have been explained to the above individual and that all questions about this donation have been answered.

____________________________________ ________________________
Signature of individual obtaining consent Date
