# Supplementary material for: The development and deployment of Common Data Elements for tissue banks for translational research in cancer – An emerging standard based approach for the Mesothelioma Virtual Tissue Bank
Source: BMC Cancer. 2008 Apr 8;8:91. doi: 10.1186/1471-2407-8-91 (PMC2329649; doi:10.1186/1471-2407-8-91)
Supplement: Additional file 2 — IRB approval letter for the Mesothelioma Virtual Bank project. [file 1471-2407-8-91-S2.pdf]

University of Pittsburgh
Institutional Review Board

3500 Fifth Avenue
Ground Level
Pittsburgh, PA 15213
(412) 383-1480
(412) 383-1508 (fax)

MEMORANDUM

TO: Michael J. Becich, M.D.

FROM: Richard Guido, M.D., Chair R Guido

DATE: September 13, 2006

SUBJECT: IRB #0608194: National Mesothelioma Virtual Registry and Tissue Bank (U19) (Mesothelioma Virtual Bank for Translational Research)

The above-referenced proposal has received expedited review and approval from the Institutional Review Board under 45 CFR 46.110 (5).

If applicable, please include the following information in the upper right-hand corner of all pages of the consent form:

Approval Date: September 13, 2006
Renewal Date: September 12, 2007
University of Pittsburgh
Institutional Review Board
IRB #0608194

Please note that it is the investigator's responsibility to report to the IRB any unanticipated problems involving risks to subjects or others [see 45 CFR 46.103(b)(5) and 21 CFR 56.108(b)]. The IRB Reference Manual (Chapter 3, Section 3.3) describes the reporting requirements for unanticipated problems which include, but are not limited to, adverse events. If you have any questions about this process, please contact the Adverse Events Coordinator at 412-383-1504.

The protocol and consent forms, along with a brief progress report must be resubmitted at least one month prior to the renewal date noted above as required by FWA00006790 (University of Pittsburgh), FWA00006735 (University of Pittsburgh Medical Center), FWA00000600 (Children's Hospital of Pittsburgh), FWA00003567 (Magee-Womens Health Corporation), FWA00003338 (University of Pittsburgh Medical Center Cancer Institute).

Please be advised that your research study may be audited periodically by the University of Pittsburgh Research Conduct and Compliance Office.

RG:kh
