# Supplementary material for: The development and deployment of Common Data Elements for tissue banks for translational research in cancer – An emerging standard based approach for the Mesothelioma Virtual Tissue Bank
Source: BMC Cancer. 2008 Apr 8;8:91. doi: 10.1186/1471-2407-8-91 (PMC2329649; doi:10.1186/1471-2407-8-91)
Supplement: Additional file 4 — Common Data Elements data dictionary with metadata and valid values. [file 1471-2407-8-91-S4.pdf]

Policy Statements on the development of Common Data Elements for annotating demographic, epidemiologic, pathology and cancer registry (outcome) data for Center for Disease Control Prevention's (CDC's) Mesothelioma Virtual Tissue Bank for Translational Research (MVB) Project

The rise of molecular biology and systems biology in medicine is driving development of bio-repositories that can provide highly annotated tissue samples to facilitate basic science, clinical as well as translational research. Clinical annotation – the association of clinical, pathologic and outcomes data with tissue samples – is central to the success of these repositories as such annotation allows samples can be better matched to the research question at hand and experimental results better understood and verified. To facilitate and standardize clinical annotation in bio-repositories, we have combined two accepted and complementary sets of data standards, the College of American Pathologists (CAP) protocol and checklist, the core elements from North American Association of Central Cancer Registry **(**NAACCR) Cancer Registry elements (for disease description, therapy and follow up) and standard epidemiological elements. Combining and extending these common approaches, - that are or soon will be mandated at most cancer centers - one can create a core set easily implemented of common data elements (CDE) for oncology tissue banking.

The purpose of the project is to develop a core set of biospecimen annotation data elements for pleural, pericardial and peritoneal mesothelioma (benign as well as malignant). We will associate these elements with a standard vocabulary system and formalize using modeling architecture to enhance both syntactic and semantic interoperability. The system will be implemented in a tissue banking database system.

We have combined the appropriate demographic, epidemiologic, pathology and cancer registry data elements to form a core set for clinical annotation of the samples. These elements include information on demographics, epidemiologic, clinical history (including therapy), pathology (at both the specimen and block level) and outcome including recurrence and vital status. The system will be implemented in a database application with a three- tiered architecture. The database is Oracle 9.2.0.1 Enterprise Edition on a SunFire V880 Server running Solaris 2.8, the middle tier is the Oracle Application Server (v 9.0.2) on a Compaq DL360 Server running Win2K. The application uses the Oracle http server and mod_plsql extensions to generate dynamic pages from the database to the users.

We will be developing the common data elements using vocabulary standards, ontology and semantic modeling methodology. The CDEs included for each case are of five different types that will include demographic data, clinical history, pathology data including block level annotation, and clinical outcome data including treatment, recurrence and vital status. These CDEs will be further enhanced to include data sets from genomics and proteomics laboratories across the participating cancer institutes to facilitate and supplement translational research. The CAP Cancer Checklist model along with the NAACCR standard will be used as the basis for an electronic data standard repository to metadata or data descriptors that will give a concrete guidance on the formulation and maintenance of discrete data element description and semantic content that shall be used to formulate data elements in a consistent and standard manner. Furthermore, this bioinformatics-driven system would eventually provide large numbers of quality-controlled tissue samples with standardized clinical and pathologic annotation for genomic and proteomic research.

Following the lead of other resources at CDC we will follow a "controlled dictionary" CDE policy. In short, every data element definition will include a data element concept (combination of class and

property or data descriptor) with associated acceptable data values (valid values) unless other wise noted. The Central Data Center will only test for and process controlled dictionary values.

Required Items:

Records submitted with missing or invalid data in any one (or more) of these fields (indicated with an asterisk (*)) will not be loaded into the database. The respective site will receive a report indicating which record(s) have been loaded and which rejected and the reason for it. Then the CDE must be corrected and resubmitted.

Conditional Required Fields:

Any Case with at least ONE SPECIMEN/BLOCK with tumor present, whether it is resection, biopsy, regional lymph node, or metastatic tissues, can qualify the case to be included in the MVB Resource. Therefore, the respective CDEs below must be filled in for the specific tissue matrix (i.e. Resection, biopsy, regional lymph nodes, or metastatic tissues) available to the resource.

Non-required Items:

Records with any errors or invalid data in non-required fields will be flagged. The respective site will receive an error report listing all case numbers with errors along with a description of the errors. Errors will be reconciled and the corrected records resubmitted within 30 days.

Submission of data files to The Central data Center:

Each local member institution's data set will be shared with the Central Data Center via one of two methods suitable to local support. One, data can be directly entered manually into the MVB Central Database. Alternatively, data files can be composed of pre-defined exported Excel files developed by the Central Data Center. The Central Data Center will process the data using the policies, variable constraints and logistical tests documented herein. Accepted records will be loaded into the central database. Unacceptable records will not be loaded into the database but returned to the submission site for review and correction. The Central Data Center will document the reasons for rejection when the unacceptable records are returned for correction.

Resubmission of records:

Only records that have been corrected should be resubmitted to the central database.

Not Available:

If data element value is not present (non-required elements only), the assumptions will be made that the data is not available. No special code need be used to define "not available". Not available is to be interpreted as unknown, not defined, not counted, etc.

Criteria for inclusion of cases

A. Any Case with at least ONE SPECIMEN / BLOCK with tumor present, whether it is resection, biopsy, regional lymph node, or metastatic tissues, can qualify the case to be included in the MVB resource.

B. **Pathology Criteria for case inclusion in MVB Frozen Tissue archive:**

- The only criteria we recommend currently are:
  1) At least 1 neoplastic block or more need to be able to be entered into the matrix or case is excluded.

C. **Priorities for entering cases in the resected specimen neoplastic block matrix:**

- These should assist the teams in picking the highest value blocks for the MVB archives.
- Since the matrix can include up to 4 blocks here are the recommendations for selection criteria:
  1) The first block should include the largest nodule of tumor (as specified by the CDE)
  2) The third and fourth blocks should include surgical margin involvement and angiolymphatic invasion (in that order of preference)
  3) The second block should include the second largest tumor.

D. **Biopsy (in cases with resection) CDE archive notes:**

- For biopsy cases, the concern is to give the reviewers some guidance on how many blocks to submit and to clarify what happens when there is only one block with tumor. In these cases it was recommended that the institution cut 3 to 4 blanks for their own diagnostic purposes and then make the residual material available to the MVB archive
- We decided that a matrix would be set up and leave the number of blocks submitted to the reviewing pathologist, encouraging them to submit as many blocks as possible on a needle biopsy to a maximum of 4. There will not be any particular order for entering blocks into the biopsy matrix.
- Following are the recommended criteria for needle biopsy (in cases with resection):
  1) Can include one block or more (up to 4).
  2) Must at least include one block and classify according to the biopsy matrix.

NOTE:

Data values highlighted in yellow indicate "Drop-down windows" and those highlighted in light blue indicate "Radio buttons/check boxes".

I. DEMOGRAPHIC and EPIDEMIOLOGIC DATA:

Many of the key patient identifiers as defined by HIPAA will not be transmitted to any research investigators. These key data sets will be managed by key Honest Brokers at each member institution and the Central Data Center.

A. Demographic Data:

1. ***MVB Number:** Electronically generated de-identified number (Enterable field).
2. ***Institutional Identification Number:** Number assigned by the originating institution.
3. **Last Name:** Last name of patient. (Enterable field).
4. **First Name:** First Name of patient. (Enterable field).
5. **Social Security Number:** Social Security number of the patient.
6. **Race:** Race of patient: White, Black, American Indian/Aleutian/Eskimo, Asian, NOS, Chinese, Japanese, Filipino, Hawaiian, Korean, Asian Indian/Pakistani, Vietnamese, Laotian, Hmong, Kampuchean, Thai, Micronesian, Chamorran, Guamanian, Polynesian, Tahitian, Samon, Tongan, Melanesian, Fiji Islander, New Guinean, Other Asian, Pacific Islander, NOS, Other, Unknown *[Cancer Registry Data Table]*.
7. **Hispanic Origin:** Code whether the patient is of Hispanic decent (Yes / No) *[Cancer Registry Data Table]*.
8. **If Yes, please specify:** Non-Spanish / Non- Hispanic, Mexican, including Chicano, Puerto Rican, Cuban, South or Central American (NOT Brazil), Other, specified Spanish or Hispanic origin, Spanish / Hispanic / Latino, NOS, Spanish surname only, Dominican Republic, Unknown.
9. ***Gender:** Sex of patient (Male / Female / Unknown).
10. **Height (centimeters):** Enterable field.
11. **Weight (kilograms):** Enterable field.
12. **Managing Physician:** From the list of Physicians name. Drop down menu. *[Cancer Registry Data Table]*.
13. **Primary Pathologist:** From the list of Pathologists. Drop down menu. *[UPMC CoPath Data Table]*.
14. **Date of Surgical Procedure:** Enterable field.
15. ***Birth date:** Record patient's date of birth in month and year found on the patient's pathology report, H&P, admitting sheet. Enterable field (MM/YEAR).
16. ***Date of first positive tissue diagnosis of mesothelioma:** Is the month and year that the primary cancer was histologically confirmed. Gold standard is biopsy date, secondary choices should include biopsy review date, and the third choice (not desirable) is the resection date if any. Enterable field (MM/YEAR).
17. **General Demographic comments:** Enterable field.

<u>B. Epidemiologic Data</u>: Data elements for genetic susceptibility to environmental carcinogens and other occupation safety and health related to mesothelioma cases.

1. **Location:** Urban / Rural / Mixed / Unknown.
2. ***Past or Present history of exposure to asbestos:** Yes/ No/ Unknown.
3. **Past or Present occupation(s):** Enterable field.
4. **History of pulmonary pathology:** Yes/ No/ Unknown.
5. **If Yes, please specify:** Enterable field.
6. ***History of smoking:**
    a. Smoker (Current or Previous) / Current / Previous / Non-smoker / Unknown.
    b. If smoker, how many years smoked? Enterable Field.
    c. If stopped, number of years since stopping? Enterable Field.
    d. Cigarettes smoked per day? Enterable Field.
    e. Packs years? Enterable Field.
7. **History of alcohol use:** Current / Previous / None / Unknown.
8. **History of other cancer:** Yes/ No/ Unknown.
9. **If yes, specify the type of cancer:** (Check boxes from the list of organ-specific cancers) Adrenal, Anus, Appendix, Bones, Breast, Brain & CNS, Colon, Cervix Uteri, Corpus Uteri, Esophagus, Gallbladder, Head & Neck, Kidney, Renal Pelvis, Ureter, Larynx, Leukemia, Liver, Lung, Lymph node, Mesothelioma, Ovary, Oral Cavity, Pancreatic, Parotid & Other Glands, Pharynx, Pleura, Prostate, Rectosigmoid, Rectum, Skin, Speen, Stomach, Testis, Thyroid, Urinary Bladder, Other, NOS, Unknown, None. *[Cancer Registry Data Table/EDRN-Colon Data Dictionary]*.
10. **Family history of cancer:** Yes/ No/ Unknown.
11. **If yes, specify the type of cancer:** (Check boxes from the list of organ-specific cancers) Adrenal, Anus, Appendix, Bones, Breast, Brain & CNS, Colon, Cervix Uteri, Corpus Uteri, Esophagus, Gallbladder, Head & Neck, Kidney, Renal Pelvis, Ureter, Larynx, Leukemia, Liver, Lung, Lymph node, Mesothelioma, Ovary, Oral Cavity, Pancreatic, Parotid & Other Glands, Pharynx, Pleura, Prostate, Rectosigmoid, Rectum, Skin, Speen, Stomach, Testis, Thyroid, Urinary Bladder, Other, NOS, Unknown, None. *[Cancer Registry Data Table/EDRN-Colon Data Dictionary]*.
12. **First degree relatives with mesothelioma: (**Check boxes from the list) Mother, Father, Sister, Brother, Half Brother, Half Sister, Son, Daughter, Grandfather, Grandmother, Unknown, None [*Cancer Registry Data Table/EDRN-Colon Data Dictionary]*.
13. **First degree relatives with any other type of cancer: (**Check boxes from the list) Mother, Father, Sister, Brother, Half Brother, Half Sister, Son, Daughter, Grandfather, Grandmother, Unknown, None [*Cancer Registry Data Table/EDRN-Colon Data Dictionary]*.
14. **Any of the non-first degree relative with mesothelioma:** Yes/ No/ Unknown.
15. ***Any Imaging study(ies) done in the patient: (**Check boxes from the list) X-ray, CT scan, MRI study, Ultrasound, Mammogram, Angiogram, V-Q scan, Radionuclide scan, Bone scan, PET scan, ERCP study, None, Unknown *[Cancer Registry Data Table/MRS]*.

16. General Epidemiologic Comment Field: Enterable.

II. SPECIMEN AVAILABLITY (INVENTORY SUMMARY):

This section will show the types of specimens available through the MVB resource.

1. **Are Mesothelioma resected specimens available?**
   Definition: Availability of mesothelioma tissue from a resected specimen.
   Data Type: Yes/No/Unknown.

2. **Type(s) of Mesothelioma resected specimens available?**
   Definition: Availability of mesothelioma tissue from a resected specimen.
   Data Type: Specify **paraffin, bulk frozen, fresh frozen tissue or glass sides.**

3. **Are Mesothelioma Biopsy specimens available?**
   Definition: Availability of biopsy tissue samples.
   Data Type: Yes/No/Unknown.

4. **Type(s) of Mesothelioma Biopsy specimens available?**
   Definition: Availability of biopsy tissue samples.
   Data Type: Specify **paraffin, bulk frozen, fresh frozen tissue or glass sides.**

5. **Are Regional Lymph Node specimens available?**
   Definition: Availability of regional lymph node tissue samples.
   Data Type: Yes/No/Unknown.

6. **Type(s) of Regional Lymph Node specimens available?**
   Definition: Availability of regional lymph node tissue samples.
   Data Type: **paraffin, bulk frozen, fresh frozen tissue or glass sides.**

7. **Are Metastatic specimens available?**
   Definition: Availability of metastatic tissue samples.
   Data Type: Yes/No/Unknown.

8. **Type(s) of Metastatic specimens available?**
   Definition: Availability of metastatic tissue samples.
   Data Type: Specify **paraffin, bulk frozen, fresh frozen tissue or glass sides.**

9. **Are Whole Blood samples available?**
   Definition: Availability of whole blood samples.
   Data Type: Yes/No/Unknown.

10. **Are Plasma samples available?**
    Definition: Availability of plasma samples.
    Data Type: Yes/No/Unknown.

11. **Are Serum samples available?**
    Definition: Availability of serum samples.
    Data Type: Yes/No/Unknown.

12. **Are Red Blood Cells (RBCs) available?**
    Definition: Availability of red blood cell samples.
    Data Type: Yes/No/Unknown.

13. **Are Peripheral Blood Mononuclear Cells (PBMCs) available?**
    Definition: Availability of peripheral blood mononuclear cells.
    Data Type: Yes/No/Unknown.

14. Are DNA samples available?

Definition: Availability of DNA samples.
Data Type: Yes/No/Unknown.

15. Are macroscopic images available?

Definition: Availability of macroscopic images.
Data Type: Yes/No/Unknown.

16 . Are microscopic images available?

Definition: Availability of microscopic images.
Data Type: Yes/No/Unknown.

III. ANATOMICAL PATHOLOGY CHARACTERISTICS:

A. Overall Histopathological Characteristics of Mesothelioma Specimen:

Enter the overall characteristics of the pathology resection specimen.

1. ***Accession Number:** Surgical Pathology Number of the specimen (Enterable).
2. ***Months between Diagnosis and Accession:** The time in months between the diagnosis (clinical) and biopsy or resection.
3. ***Type of procedure:** Name of the surgical procedure used to obtain the surgical pathology specimen of mesothelioma (Core needle biopsy, percutaneous needle biopsy, incisional biopsy, thoracoscopic biopsy, biopsy not specified, open thoracotomy, pleural resection, pneumonectomy, pericardial resection, peritoneal resection, lymph node resection, lymph node biopsy, other, not specified).
4. ***Site of specimen:** Anatomic localization of the tumor (Right visceral pleura, left visceral pleura, right parietal pleura, left parietal pleura, diaphragmatic pleura, right pleura, left pleura, pleura, pericardium, peritoneum, pelvic peritoneum, metastasis, other, not specified).
5. **Site of metastasis:** Enterable Field.
6. **Invasive tumor present:** Was invasive tumor identified in the accession? (Yes/No/Unknown/Not applicable).
7. ***Primary or metastatic tumor:** (Primary/Metastasis/Not specified)
8. ***Date of Resection or Biopsy:**
   Definition: Record patient's date of resection in month and year found on the patient's pathology report, H&P, admitting sheet.
   Data Type: Integer: MM/YEAR

9. Tumor Size:

Tumor size can be determined: (Yes/No)
Greatest dimension (cm): Greatest length (in centimeters) of all the three dimensions.
Additional dimensions (cm): Second dimension length in centimeters.
Maximum thickness (cm): Maximum thickness in centimeters.

10. ***Most Prominent Histological Type of Invasive Cancer:**

Definition: Most prominent overall histological type present in the specimen.
User entered text indicating a diagnostic sub classification of the type of mesothelioma (Epithelial or epithelioid mesothelioma, Sarcomatoid mesothelioma, Biphasic mesothelioma, Multicystic mesothelioma, Papillary mesothelioma, Desmoplastic mesothelioma, Other Mesothelioma, type not specified, Unknown).
Data Type: Character String.

11. ***Tumor Differentiation or Grade:**

Definition: Grade or degree of differentiation of the tumor (High, Intermediate, Low, Indeterminate, Not specified, Not applicable).
Data Type: Character String.

12. Size of Largest Individual Nodule of Cancer (in cm):

Definition: Size of largest individual nodule of invasive cancer present in the resected or biopsy specimen.

Data Type: Double (number).

13. ***Tumor configuration:***

Definition: Configuration or the area occupied by the tumor cells within the tissue of origin. (Exophytic, Endophytic, Nodular, Papillary, Cystic, Mixed, Diffuse, Localized, Not specified).

14. Extent of invasion:

Definition: Extent (local or distant) of spread of the tumor away from the tissue of origin. (Lung parenchyma, Endothoracic fascia, Contralateral pleura, Soft tissue of chest wall, Rib, Vertebra, Brachial plexus, Mediastinum, Pericardium, Myocardium, Diaphragm, Peritoneum, Omentum, Abdominal viscera, Pelvic viscera, Other, Cannot be determined, Not applicable)

15. Surgical Margin (SM) Involvement

Definition: Are Surgical Margins involved by Tumor (ink on margin)?
Data Type: Character String (Yes/No/Unknown/Not applicable).

16. Presence of Angiolymphatic (AL) Invasion:

Definition: Is Large and /or small vessel Invasion present in the resected specimen?
Data Type: Character String (Yes/No/Unknown/Not applicable).

17. Extrapleural or extraperitoneal or extrapericardial extension:

Definition: Presence or absence of extrapleural or extraperitoneal or extrapericardial extension of the tumor.

Data Type: Yes/No/Unknown/Not applicable.

18. Additional Pathologic Findings:

Definition: Histopathological findings other than the tumor as described in the pathology report. (Ferruginous bodies, Pleural plaque, Pulmonary interstitial fibrosis, Acute inflammation, Chronic inflammation, Mixed inflammation, Changes of talc pleurodesis, None identified, Other).

19. Histochemical Special Stain Profile:

Definition: Histochemical/special staining characterization of mesothelioma as described in the pathology report.
Data Type: Character String.

20. Immunohistochemical Profile:

Definition: Immunohistochemical staining characterization of mesothelioma as described in the pathology report.
Data Type: Character String.

21. Ultrastructural Findings:

Definition: Ultrastructural/Electron microscopic findings of mesothelioma as described in the pathology report.
Data Type: Character String.

22. General Comments Section on resection/biopsy:

Definition: General comments related to resection.
Data Type: Character String.

B. Neoplastic Paraffin Block Matrix:

(These are multiple entry fields. Up to 4 blocks preferred)

The Paraffin block matrix section will allow the collection of block details on cases that meet the minimum requirements for inclusion into the resource. NOTE: Priorities for entering cases in resected mesothelioma block matrix.

- **These should assist the teams in picking the highest value blocks for the MVB archives.**
- **Since the matrix can include up to 4 blocks here are the recommendations for selection criteria:**
  1) **The *first block* should include the largest nodule of tumor (as specified by the CDE)**
  2) **The *third and fourth blocks* should include surgical margin involvement (SM) or angiolymphatic invasion (AL) (in that order of preference).**
     NOTE: If SM and AL do not occur, select the next largest area of tumor for 3 and 4 block of the matrix.
  3) **The *second block* should include the second largest tumor.**

1. **Block Matrix: Type of Block(s) available**
   Definition: Are frozen, paraffin, or both types of blocks available?
   Data Type: Character String (Paraffin/Frozen/Both).

2. **Block Matrix: Accession number**
   Definition: Surgical Pathology Number of the specimen.
   Data Type: Character String.

3. **Paraffin Block Matrix: Block number**
   Definition: Block number of the block with largest tumor nodule.
   Data Type: Character String.

4. **Paraffin Block Matrix: Most Prominent Histological Type**
   Definition: Most prominent histological type present on block.
   User entered text indicating a diagnostic sub classification of the type of mesothelioma (Epithelial or epithelioid mesothelioma, Sarcomatoid mesothelioma, Biphasic mesothelioma, Multicystic mesothelioma, Papillary mesothelioma, Desmoplastic mesothelioma, Other Mesothelioma, type not specified, Unknown).
   Data Type: Character String.

5. **Paraffin Block Matrix: Size of Largest Individual Nodule of Cancer (in cm)**
   Definition: Size of cancer present in block.
   Data Type: Double (number).

6. **Paraffin Block Matrix: Presence of Positive Surgical Margin (SM)**
   Definition: Positive surgical margin in block.
   Data Type: Yes/No/Unknown/Not applicable.

7. **Paraffin Block Matrix: Presence of Angiolymphatic Invasion (AL):**
   Definition: Presence of large and / or small vessel Invasion in block.
   Data Type: Yes/No/Unknown/Not applicable.

8. **Paraffin Block Matrix: Block comments**
   Definition: Comments related to paraffin block matrix.
   Data Type: Character String.

C. Non-neoplastic Paraffin Blocks:

Try to include at least one block of normal lung if possible (two blocks are preferred). If it is not possible to find a completely normal block then include one with minimal amounts of mesothelioma tissue.

1. **Paraffin resection specimen Non-Neoplastic Block #1:**
   Definition: Block number of non-neoplastic block #1
   Data Type: Character String.
2. **Paraffin resection specimen Non-Neoplastic Block #2:**
   Definition: Block number of non-neoplastic block #2
   Data Type: Character String.
3. **General Comments on Paraffin Blocks:**
   Definition: General comments related to all paraffin blocks.
   Data Type: Character String.

D. Neoplastic Frozen Bulk Block Matrix:

(These are multiple entry fields. Up to 4 blocks preferred)
Same as above.
NOTE: This section is for the FROZEN BLOCK matrix.
It is very possible that some (and occasionally all) of the frozen blocks in the matrix will also have paraffin tissue. When this happens indicate this in data element [Type of Block(s) available]. On occasion, there may be some blocks that are only frozen (site dependent) also indicate this in the aforementioned data items.

1. **Block Matrix: Type of Block(s) available**
   Definition: Are frozen, paraffin, or both types of blocks available?
   Data Type: Character String (Paraffin/Frozen/Both).
2. **Frozen Tissue: Warm Ischemic Time (in minutes)**
   Definition: Turn around time of when tissue was removed from the patient and the time when it was banked.
   Data Type: Double (number).
3. **Frozen Block Matrix: Block number**
   Definition: Block number of the block with largest tumor nodule.
   Data Type: Character String.
4. **Frozen Block Matrix: Most Prominent Histological Type**
   Definition: Most prominent histological type present on block. User entered text indicating a diagnostic sub classification of the type of mesothelioma (Epithelial or epithelioid mesothelioma, Sarcomatoid mesothelioma, Biphasic mesothelioma, Multicystic mesothelioma, Papillary mesothelioma, Desmoplastic mesothelioma, Other Mesothelioma, type not specified, Unknown).
   Data Type: Character String.
5. **Frozen Block Matrix: Size of Largest Individual Nodule of Cancer (in cm)**
   Definition: Size of cancer present in block.
   Data Type: Double (number).

6. **Frozen Block Matrix: Presence of Positive Surgical Margin (SM)**
   Definition: Positive surgical margin in block.
   Data Type: Yes/No/Unknown/Not applicable.

7. **Frozen Block Matrix: Presence of Angiolymphatic Invasion (AL)**
   Definition: Presence of large and / or small vessel Invasion in block.
   Data Type: Yes/No/Unknown/Not applicable.

8. **Frozen Block Matrix: Block comments**
   Definition: Comments related to frozen block matrix.
   Data Type: Character String.

E. Non-neoplastic Bulk Frozen Blocks:

Try to include at least one block of normal lung if possible (two blocks are preferred). If it is not possible to find a completely normal block then include one with minimal amounts of mesothelioma tissue.

1. **Frozen Resection specimen Non-Neoplastic Block #1:**
   Definition: Block number of non-neoplastic block #1
   Data Type: Character String.

2. **Frozen Resection specimen Non-Neoplastic Block #2:**
   Definition: Block number of non-neoplastic block #2
   Data Type: Character String.

3. **General Comments on Frozen Blocks:**
   Definition: General comments related to all frozen blocks.
   Data Type: Character String.

F. Biopsy Block Matrix:

The biopsy block matrix section will allow the collection of block details on biopsy samples that meet the minimum requirements for inclusion into the Resource.

- For biopsy cases, the concern is to give the reviewers some guidance on how many blocks to submit and to clarify what happens when there is only one block with tumor. In these cases it will be recommended that the institution cut 3 to 5 blanks for their own diagnostic purposes and then make the residual material available to the MVB archive.
- A matrix block will be set up and leave the number of blocks submitted to the reviewing pathologist, encouraging them to submit as many blocks as possible on a biopsy to a maximum of 5. There will be no particular order for entering blocks into the biopsy matrix.
- The recommended criteria for biopsy will be as follows:
  1) Can include one block or more (up to 5)
  2) Must at least include one neoplastic block and classify according to the biopsy matrix.
  NOTE: for biopsy-only cases, it is a requirement to enter the "subsequent resection" field in the biopsy attribute section.

1. **Biopsy Matrix: Block number**
   Definition: Block number of the block with largest tumor nodule.
   Data Type: Character String.

2. **Biopsy Matrix: Most Prominent Histological Type of Invasive Cancer**
   Definition: Most prominent histological type present on block. User entered text

indicating a diagnostic sub classification of the type of mesothelioma (Epithelial or epithelioid mesothelioma, Sarcomatoid mesothelioma, Biphasic mesothelioma, Multicystic mesothelioma, Papillary mesothelioma, Desmoplastic mesothelioma, Other Mesothelioma, type not specified, Unknown).

Data Type: Character String.

3. **Biopsy Matrix: Grade**

Definition: Grade or degree of differentiation of the tumor (High, Intermediate, Low, Indeterminate, Not specified, Not applicable).

Data Type: Character String.

4. **Biopsy Matrix: Size of Largest Individual Nodule of Invasive Cancer (in cm)**

Definition: Size of invasive cancer present in block.

Data Type: Double (number).

5. **Biopsy Matrix: Presence of Angiolymphatic Invasion (AL)**

Definition: Presence of Angiolymphatic Invasion in block.

Data Type: Yes/No/Unknown/Not applicable.

6. **Biopsy Matrix: Block comments**

Definition: Comments related to paraffin block matrix.

Data Type: Character String.

G. Regional Lymph Node Status at the time of resection:

Include the lymph node status at the time of the resection.

-If there is regional lymph node exploration prior or equal to the resection date, but after the initial diagnostic biopsy, then enter values here (in the Lymph Node section).

-Regional lymph nodes removed after the resection of tumor or Distant lymph nodes should be enter in the *METASTASIS MATRIX* section.

1. **Date of Regional Lymph Node resection**

Definition: Record patient's date of regional lymph node resection month and year found on the patient's pathology report, H&P, admitting sheet.

Data Type: Integer: MM/YYYY

2. **Number of Nodes Examined**

Definition: Number of lymph nodes examined at the time of the resection.

Data Type: Integer (number).

3. **Number of Nodes Positive**

Definition: Number of positive lymph nodes examined at the time of the resection.

Data Type: Integer (number).

4. **Lymph Node Non-Neoplastic Block #1:**

Definition: Block number of non-neoplastic block #1

Data Type: Character String.

5. **Lymph Node Block Matrix: Neoplastic Block number:**

Definition: Block number of metastatic lymph node block

Data Type: Character String.

6. **Lymph Node Block Matrix: Size of Largest Individual Nodule of Cancer (in cm)**

Definition: Size of invasive cancer present in lymph node block.

Data Type: Double (number).

7. **Lymph Node Block Matrix: Presence of Extracapsular Extension (ECE)**
   Definition: Presence of Extracapsular Extension in block.
   Data Type: Yes/No/Unknown.

8. **General Comments Section for Lymph Nodes:**
   Definition: General comments related to resection.
   Data Type: Character String.

H. Metastatic Tissue Block Matrix:

-Enter as many blocks available (Three blocks are preferred).
-If multiple metastatic sites are present, then enter at least 1 block from each site.
-Try to include at least one block of normal tissue from the same site if possible. If it is not possible to find a completely normal block then include one with minimal amounts of tumor.

1. **Metastatic Block Matrix: Type of Block(s) available**
   Definition: Type of metastatic block available.
   Data Type: Character String (Paraffin/Frozen/Both).

2. **Date of Metastatic Block Matrix:**
   Definition: Date when metastatic tissue was removed/excised.
   Data Type: Integer: MM/YYYY

3. **Metastatic Block Matrix: Block number**
   Definition: Block number of the block with largest tumor nodule in the metastatic tissue.
   Data Type: Character String.

4. **Metastatic Block Matrix: Specimen source**
   Definition: Procedure/Method used to acquire specimen.
   Data Type: Character String (Resection/Biopsy/Both/Not specified).

5. **Metastatic Block Matrix: Most Prominent Histological Type of Invasive Cancer**
   Definition: Most prominent histological type present on metastatic block. User entered text indicating a diagnostic sub classification of the type of mesothelioma (Epithelial or epithelioid mesothelioma, Sarcomatoid mesothelioma, Biphasic mesothelioma, Multicystic mesothelioma, Papillary mesothelioma, Desmoplastic mesothelioma, Other Mesothelioma, type not specified, Unknown).
   Data Type: Character String.

6. **Metastatic Block Matrix: Size of Largest Individual Nodule of Invasive Cancer (in cm)**
   Definition: Size of invasive cancer present in metastatic block.
   Data Type: Double (number).

7. **Metastatic Block Matrix: Presence of Therapy effects**
   Definition: Therapy effect: hormonal/radiation effect on tumor.
   Data Type: Character String (Surgery, Radiation therapy, Chemotherapy, Hormone therapy, Immunotherapy, Brachytherapy, Cryotherapy, External beam Watchful expectancy, Other, None).

8. **Metastatic Block Matrix: Non-Neoplastic Block number:**
   Definition: Block with non-neoplastic tissue associated with the metastatic block

submitted above.
Data Type: Character String.

9. General Comments Section for Metastatic Block Matrix:
Definition: General comments related to metastatic specimens.
Data Type: Character String.

IV. GENOTYPES DATA:

1. **Genotype Data Available:**
   Definition: Availability of genotype data
   Data Type: Yes/No/Unknown/Not Accessed

2. **GSTM1 Genotype:**
   Definition: Glutathione S-transferase M1 genotype
   Data Type: Functional (0)/Null (2)

3. **GSTT1 Genotype:**
   Definition: Glutathione S-transferase T1 genotype
   Data Type: Functional (0)/Null (2)

4. **CYP1A1 Genotype:**
   Definition: Cytochrome P450 1A1 genotype
   Data Type: Homozygous wild-type (0)/Heterozygous (1)/Homozygous variant (2)

5. **NAT2 Genotype:**
   Definition: N- acetyltransferase 2 genotype
   Data Type: Slow (0)/ Fast (1)

6. **EPHX1 Genotype:**
   Definition: Microsomal epoxide hydrolase 1 (EPHX1) gene activity
   Data Type: High (0)/Intermediate (1)/Low (2)

7. **EPHX3 Genotype:**
   Definition: EPHX1 exon 3 polymorphism
   Data Type: Homozygous wild-type (0)/Heterozygous (1)/Homozygous variant (2)

8. **EPHX4 Genotype:**
   Definition: EPHX1 exon 4 polymorphism
   Data Type: Homozygous wild-type (0)/Heterozygous (1)/Homozygous variant (2)

V. STAGING:

Pathological and Clinical Staging will be based on using the AJCC Manual for Staging of Cancer.

A. AJCC Manual for Staging of Cancer:

1. **AJCC Manual Edition Number:**
   Definition: Which edition of the AJCC Manual for Staging of Cancer was used for pathological/clinical staging evaluation?
   Data Type: Character String (1st, 2nd, 3rd, 4th, 5th, 6th, , Not applicable, Unknown).

B. Pathological Staging:

1. **pT Stage**
   Definition: Use AJCC Manual for Staging of Cancer, Report pT stage at initial diagnosis.
   Data Type: Character String (TX, T0, T1a, T1b, T2, T3, T4, Not applicable, Unknown).

2. **pN Stage**
   Definition: Use AJCC Manual for Staging of Cancer, Report pN stage at initial diagnosis.
   Data Type: Character String (NX, N0, N1, N2, N3, Not applicable, Unknown).

3. **pM Stage**
   Definition: Use AJCC Manual for Staging of Cancer, Report pM stage at initial diagnosis.
   Data Type: Character String (MX, M0, M1, Not applicable, Unknown).

C. Clinical Staging:

1. **cT Stage**
   Definition: Use AJCC Manual for Staging of Cancer. Report cT stage at initial diagnosis.
   Data Type: Character String (TX, T0, T1, T2, T3, T4, Not applicable, Unknown).

2. **cN Stage**
   Definition: Use AJCC Manual for Staging of Cancer. Report cN stage at initial diagnosis.
   Data Type: Character String (NX, N0, N1, N2, N3, Not applicable, Unknown).

3. **cM Stage**
   Definition: Use AJCC Manual for Staging of Cancer. Report cM stage at initial diagnosis.
   Data Type: Character String (MX, M0, M1, Not applicable, Unknown).

4. **General Staging Comments**
   Definition: General Comments related to Staging (Pathological and Clinical).
   Data Type: Character String.

VI. THERAPY RELATED DATA:

Record the patient's treatment history.

1. *__Therapy matrix: Type of Therapy:__ Surgery, Radiation therapy, Chemotherapy, Hormone therapy, Immunotherapy, Brachytherapy, Cryotherapy, External beam, Watchful expectancy, Other, and None.
   Definition: Report type of therapy patient was given.
   Data Type: Character String.

2. **Therapy matrix: Therapy Start Date**
   Definition: Enter the date when patient started treatment.
   Data Type: Integer: MM/YYYY

3. **Presence of pleural effusion:**
   Definition: Presence of absence of pleural effusion.
   Data Type: Character String (Yes/No/Unknown).

4. **Presence of ascites:**
   Definition: Presence of absence of ascites.
   Data Type: Character String (Yes/No/Unknown).

5. **Comments on Therapy response:** Enterable Field.

6. **General Overall Comments on Therapy:** Enterable Field.

VII. CLINICALLY VERIFIED TISSUE RECURRENCE / METASTASIS DATA:

Verification for clinical recurrence can be via radiology imaging, biopsy/resection, surgery, or cancer registry. However, a clinician's note indicating recurrence in a specific distant site would be sufficient.

1. **Date of Tissue Recurrence/Metastasis: Distant site 1**
   Definition: Date when tissue recurrence is clinically verified and documented in the medical chart.
   Data Type: Integer: MM/YYYY

2. **Tissue Recurrence/Metastasis: Distant site 1**
   Definition: Location of clinically verified tissue recurrence (site 1).
   Data Type: Character String (Adrenal, Anus, Appendix, Bones, Breast, Brain & CNS, Colon, Cervix Uteri, Corpus Uteri, Esophagus, Gallbladder, Head & Neck, Kidney, Renal Pelvis, Ureter, Larynx, Leukemia, Liver, Lung, Lymph node, Mesothelioma, Ovary, Oral Cavity, Pancreatic, Parotid & Other Glands, Pharynx, Pleura, Prostate, Rectosigmoid, Rectum, Skin, Speen, Stomach, Testis, Thyroid, Urinary Bladder, Other, NOS, Unknown, None) *[Cancer Registry Data Table/EDRN-Colon Data Dictionary]*.

3. **Date of Tissue Recurrence/Metastasis: Distant site 2**
   Definition: Date when tissue recurrence is clinically verified and documented in the medical chart.
   Data Type: Integer: MM/YYYY

4. **Tissue Recurrence/Metastasis: Distant site 2**
   Definition: Location of clinically verified tissue recurrence (site 2).
   Data Type: Character String (Adrenal, Anus, Appendix, Bones, Breast, Brain & CNS, Colon, Cervix Uteri, Corpus Uteri, Esophagus, Gallbladder, Head & Neck, Kidney, Renal Pelvis, Ureter, Larynx, Leukemia, Liver, Lung, Lymph node, Mesothelioma, Ovary, Oral Cavity, Pancreatic, Parotid & Other Glands, Pharynx, Pleura, Prostate, Rectosigmoid, Rectum, Skin, Speen, Stomach, Testis, Thyroid, Urinary Bladder, Other, NOS, Unknown, None) *[Cancer Registry Data Table/EDRN-Colon Data Dictionary]*.

5. **Date of Tissue Recurrence/Metastasis: Distant site 3**
   Definition: Date when tissue recurrence is clinically verified and documented in the medical chart.
   Data Type: Integer: MM/YYYY

6. **Tissue Recurrence/Metastasis: Distant site 3**
   Definition: Location of clinically verified tissue recurrence (site 3).
   Data Type: Character String (Adrenal, Anus, Appendix, Bones, Breast, Brain & CNS, Colon, Cervix Uteri, Corpus Uteri, Esophagus, Gallbladder, Head & Neck, Kidney, Renal Pelvis, Ureter, Larynx, Leukemia, Liver, Lung, Lymph node, Mesothelioma, Ovary, Oral Cavity, Pancreatic, Parotid & Other Glands, Pharynx, Pleura, Prostate, Rectosigmoid, Rectum, Skin,

Speen, Stomach, Testis, Thyroid, Urinary Bladder, Other, NOS, Unknown, None) *[Cancer Registry Data Table/EDRN-Colon Data Dictionary]*.

7. **General Comments for Clinically Verified Tissue Recurrence/Metastasis:**
   Definition: General comments related to clinically verified recurrence/metastasis.
   Data Type: Character String

VIII. VITAL STATUS / FOLLOW UP DATA:

Record the patient's vital status and most recent follow up date.

Vital Status/Follow Up:

1. ***Vital Status:**
   Definition: Using the most accurate information available, this could include the patient's medical chart, a physician chart, another tumor registry, record patient vital status.

   Data Type: Character String (Alive, Dead, Unknown).

2. ***Cancer Status:** Evidence of cancer, No evidence of cancer, Unknown.

3. ***Date Last Known Alive:**
   Definition: Date should be linked to when the last follow up was done.
   Data Type: Integer: MM/YYYY

4. **Date of Death:**
   Definition: Enter the date when patient is known to have died.
   Data Type: Integer: MM/YYYY

5. **Cause of Death:** Directly, Indirectly, Not caused by cancer, Unknown.

6. **Final Comments:**
   Definition: General final comments related to this patient.
   Data Type: Character String.
