# Supplementary material for: The development and deployment of Common Data Elements for tissue banks for translational research in cancer – An emerging standard based approach for the Mesothelioma Virtual Tissue Bank
Source: BMC Cancer. 2008 Apr 8;8:91. doi: 10.1186/1471-2407-8-91 (PMC2329649; doi:10.1186/1471-2407-8-91)
Supplement: Additional file 5 — Data collection template for the Mesothelioma Virtual Bank project. [file 1471-2407-8-91-S5.pdf]

MVB Common Data Elements for Collection of Mesothelioma Specimens PAPER FORMS [Based on Data Dictionary Version 09/20/07 – Additional File # 4]

USER INSTRUCTIONS

Form Completion Guidelines:

1) Priorities for completing the form are to fill out all required elements followed by any subset of data that will allow for inclusion in the MVB databank.
2) Any corrections of changes to the forms should be e-mailed to Nancy Whelan (whelanb@upmc.edu).

STEPS involved in initial screening, inclusion and classification of MVB cases

STEP 1 - Pre-screening cases (Research Assistant's Role)

Required Data Elements Pre-Check: These data element searches are recommended before pathologist review. Make sure the case has the following REQUIRED data elements before beginning microscopic examination:

- **At least 1 blocks are available on case that include mesothelioma *or* 1 block for biopsy-only or metastatic cases.**
- **Gender, Date of Birth, Date of Diagnosis, Asbestos Exposure History, and Vital Status must be available to qualify a case and should be collected BEFORE microscopic exam and central review by the pathologist is initiated.**
- **Also # Nodes Examined/Positive must be available.**

NOTE: If these data elements are not found, make a record of this case this for later discussion. We must determine the exclusion rate and if too high we may need modified "required" (asterisked) data elements.

STEP 2 - Pathologist Exam

The "pre-screened case" is given to pathologist who begins data recording (Pathologist's Role).

Pathologist determines priorities for entering cases in resected mesothelioma block matrix (Pathologist's Role)

- **Priorities for entering cases in neoplastic block matrix**
- **These should assist the teams in picking the highest value blocks for the MVB archives.**
- **Since the matrix can include up to 4 blocks here are the recommendations for selection criteria:**
  1) **The first block should include the largest nodule of tumor (as specified by the CDE).**
  2) **The third through fourth blocks should include surgical margin involvement (SM) or angiolymphatic invasion (AL) [in that order of preference].**
  3) **The second block should include the second largest tumor.**

4) Additionally, try to include at least one block of normal lung if possible (two blocks are preferred). If it is not possible to find a completely normal block, then include one with minimal amounts of mesothelioma tumor.

STEP 3 - Clinical Information Collection (Research Assistant's Role)

If possible, fill out the remaining parts of the form and include the time it took to complete this subsection.

STEP 4 - Biopsy CDE guidelines (Pathologist's Role)

For biopsy cases, a matrix similar to the resection was developed and the criteria are:

1) Can include one block or more (up to a maximum of 5).
2) Must at least include one neoplastic block and classify according to the biopsy matrix.
3) Try to submit as many blocks as possible.

STEP 5 - Frozen Tissue archive guidelines (Pathologist's Role)

- Some of the frozen blocks in the matrix will also have paraffin tissue – please indicate this in the data element [Type of Block(s) Available].
- If there are only frozen blocks (site dependent), then please indicate this as described above.
- Try to include at least one block of normal lung if possible (two blocks are preferred). If it is not possible to find a completely normal block, then include one with minimal amounts of mesothelioma tumor.

STEP 6 – Lymph Node / Metastasis CDE guidelines (Pathologist's Role).

For lymph node and metastatic cases, the criteria are:

- Enter regional lymph node explorations prior to or equal to the resection date (but after the initial diagnostic biopsy) in the Lymph Node Block Matrix.
- Regional lymph nodes removed AFTER the resection of tumor, or distant lymph nodes should be entered in the Metastatic Tissue Block Matrix. Can include one block or more (up to a maximum of 5):
    1) Enter as many blocks available (Three blocks are preferred)
    2) If mutiple metastatic sites are present, then enter at least 1 block from each site.
    3) Try to include at least 1 block of normal tissue from the same site if possible (or a block with minimal amounts of tumor).

DEMOGRAPHICS - INTERNAL REFERENCE DATA

(This section is NOT to be supplied to MVB Central Data Center - only for internal record keeping use only and NOT to be passed on to Central Data Center)

*1. MVB Number: ____

*2. Institutional Identification Number ___________________________

3. Last Name: ____

4. First Name: ____

5. Social Security Number: __ __ __ - __ __ - __ __ __ __

DEMOGRAPHIC DATA

*1. **MVB Number:** ____

2. **Race:** (check one)

☐ White	☐ Vietnamese	☐ Samoan
☐ Black	☐ Laotian	☐ Tongan
☐ American Indian/Aleutian/Eskimo	☐ Hmong	☐ Melanesian
☐ Asian, NOS	☐ Kampuchean	☐ Fiji Islander
☐ Chinese	☐ Thai	☐ New Guinean
☐ Japanese	☐ Micronesian	☐ Other Asian
☐ Filipino	☐ Chamorran	☐ Pacific Islander, NOS
☐ Hawaiian	☐ Guamanian	☐ Other
☐ Korean	☐ Polynesian	☐ Unknown
☐ Asian Indian/Pakistani	☐ Tahitian	

3. **Hispanic Origin:** (circle one) **Yes** **No**

If Yes, please specify: (check one)

☐ Non-Spanish/Non-Hispanic	☐ Other, specified Spanish or Hispanic origin
☐ Mexican, including Chicano	☐ Spanish/Hispanic/Latino, NOS
☐ Puerto Rican	☐ Spanish surname only
☐ Cuban	☐ Dominican Republic
☐ South or Central America (NOT Brazil)	☐ Unknown

*4. **Gender:** (circle one) **Male** **Female** **Unknown**

5. **Height:** __ __ __ cm

6. **Weight:** __ __ __ kg

7. **Managing Physician:** ____

8. **Primary Pathologist:** ____

9. **Date of Surgical procedure:** __ __ /__ __ __ __ (MM/YYYY)

*10. **Birth date:** __ __ / __ __ __ __ (MM/YYYY)

*11. **Date of First Positive Tissue Diagnosis of Mesothelioma:** __ __ /__ __ __ __ (MM/YYYY)

12. **General Demographic Comments:** ____________________________________
____
____
____

EPIDEMIOLOGIC DATA

Data elements for genetic susceptibility to environmental carcinogens and other occupation safety and health related to mesothelioma cases.

1. **Location:** (circle one) Urban Rural Mixed Unknown

*2. **Past or Present History of Exposure to Asbestos:** (circle one)
Yes No Unknown

3. **Past or Present Occupation(s):** ______________________________________
____
____
____

4. **History of Pulmonary Pathology:** (circle one) Yes No Unknown
If Yes, please specify: ____
____
____

*5. **History of Smoking:**
a. Circle one: Smoker (Current or Previous) Current smoker Previous smoker Non-smoker Unknown
b. If smoker, how many years smoked? _____
c. If stopped, # of years since stopping? _____
d. Cigarettes smoked per day? _____
e. Pack years? _____

6. **History of Alcohol Use:** (circle one) Current Previous None Unknown

7. **History of Other Cancer:** (circle one) Yes No Unknown

8. **If Yes, please specify the type of cancer:** (check all that apply)

☐ Adrenal	☐ Larynx	☐ Rectosigmoid
☐ Anus	☐ Leukemia	☐ Rectum
☐ Appendix	☐ Liver	☐ Skin
☐ Bones	☐ Lung	☐ Spleen
☐ Breast	☐ Lymph node	☐ Stomach
☐ Brain & CNS	☐ Mesothelioma	☐ Testis
☐ Colon	☐ Ovary	☐ Thyroid
☐ Cervix Uteri	☐ Oral Cavity	☐ Urinary Bladder
☐ Corpus Uteri	☐ Pancreatic	☐ Other, NOS
☐ Esophagus	☐ Parotid & Other Glands	☐ Unknown
☐ Gallbladder	☐ Pharynx	☐ None
☐ Head & Neck	☐ Pleura	☐
☐ Kidney, Renal Pelvis, Ureter	☐ Prostate	

9. **Family History of Cancer:** (circle one) Yes No Unknown

10. **If Yes, please specify the type of cancer:** (check all that apply)

☐ Adrenal	☐ Larynx	☐ Prostate
☐ Anus	☐ Leukemia	☐ Rectosigmoid
☐ Appendix	☐ Liver	☐ Rectum
☐ Bones	☐ Lung	☐ Skin
☐ Breast	☐ Lymph node	☐ Spleen
☐ Brain & CNS	☐ Mesothelioma	☐ Stomach
☐ Colon	☐ Ovary	☐ Testis

- ☐ Cervix Uteri
- ☐ Corpus Uteri
- ☐ Esophagus
- ☐ Gallbladder
- ☐ Head and Neck
- ☐ Kidney, Renal Pelvis, Ureter
- ☐ Oral Cavity
- ☐ Pancreatic
- ☐ Parotid & Other Glands
- ☐ Pharynx
- ☐ Larynx
- ☐ Pleura
- ☐ Thyroid
- ☐ Urinary Bladder
- ☐ Other, NOS
- ☐ Unknown
- ☐ None

11. First Degree Relatives with Mesothelioma: (check all that apply)

- ☐ Mother
- ☐ Father
- ☐ Sister
- ☐ Brother
- ☐ Half Brother
- ☐ Half Sister
- ☐ Son
- ☐ Daughter
- ☐ Grandfather
- ☐ Grandmother
- ☐ Unknown
- ☐ None

12. First Degree Relatives with Any Other Type of Cancer: (check all that apply)

- ☐ Mother
- ☐ Father
- ☐ Sister
- ☐ Brother
- ☐ Half Brother
- ☐ Half Sister
- ☐ Son
- ☐ Daughter
- ☐ Grandfather
- ☐ Grandmother
- ☐ Unknown
- ☐ None

13. Any Non-first Degree Relatives with Mesothelioma: (circle one)

Yes No Unknown

***14. Any Imaging Study(ies) Done in the Patient? (check all that apply)**

- ☐ X-ray
- ☐ CT scan
- ☐ MRI study
- ☐ Ultrasound
- ☐ Mammogram
- ☐ Angiogram
- ☐ V-Q scan
- ☐ Radionuclide scan
- ☐ Bone scan
- ☐ PET scan
- ☐ ERCP study
- ☐ None
- ☐ Unknown

15. General Epidemiologic Comments: ____________________________________

____

____

____

Specimen Availability (Inventory Summary)

This section will show the types of specimens available through the MVB resource.

1. **Are Mesothelioma Resected specimens available?** (circle one)

   Yes No Unknown

2. **Type(s) of Mesothelioma Resected specimens available:** (check all that apply)

   ☐ Paraffin ☐ Bulk Frozen ☐ Fresh Frozen ☐ Glass Slides

3. **Are Mesothelioma Biopsy specimes available?** (circle one)

   Yes No Unknown

4. **Type(s) of Mesothelioma Biopsy specimens available:** (check all that apply)

   ☐ Paraffin ☐ Bulk Frozen ☐ Fresh Frozen ☐ Glass Slides

5. **Are Regional Lymph Node specimens available?** (circle one)

   Yes No Unknown

6. **Type(s) of Regional Lymph Node specimens available:** (check all that apply)

   ☐ Paraffin ☐ Bulk Frozen ☐ Fresh Frozen ☐ Glass Slides

7. **Are Metastatic specimens available?** (circle one)

   Yes No Unknown

8. **Type(s) of Metastatic specimens available:** (check all that apply)

   ☐ Paraffin ☐ Bulk Frozen ☐ Fresh Frozen ☐ Glass Slides

9. **Are Whole Blood samples available?** (circle one)

   Yes No Unknown

10. **Are Plasma samples available?** (circle one)

    Yes No Unknown

11. **Are Serum samples available?** (circle one)

    Yes No Unknown

12. **Are Red Blood Cells (RBCs) available?** (circle one)

    Yes No Unknown

13. **Are Peripheral Blood Mononuclear Cells (PBMCs) available?** (circle one)

    Yes No Unknown

14. **Are DNA samples available?** (circle one)

    Yes No Unknown

15. **Are Macroscopic images available?** (circle one)

    Yes No Unknown

16. **Are Microscopic images available?** (circle one)

    Yes No Unknown

ANATOMICAL PATHOLOGY CHARACTERICTICS

Overall Histopathological Characteristics of Mesothelioma Specimen:

Enter the overall characteristics of the pathology resection specimen.

*1. **Accession Number** (i.e. SP-91-645): __ __ __ - __ __ - __ __ __ __ __

*2. **Months Between Diagnosis and Accession:** _____ **months**

*3. **Type of Procedure:** (check all that apply)

☐ Core needle biopsy	☐ Open thoracotomy	☐ Lymph node resection
☐ Percutaneous needle biopsy	☐ Pleural resection	☐ Lymph node biopsy
☐ Incisional biopsy	☐ Pneumonectomy	☐ Other
☐ Thoracoscopic biopsy	☐ Pericardial resection	☐ Not Specified
☐ Biopsy, not specified	☐ Peritoneal resection	

*4. **Site of Specimen:** (check all that apply)

☐ Right visceral pleura	☐ Right pleura	☐ Pelvic peritoneum
☐ Left visceral pleura	☐ Left pleura	☐ Metastasis
☐ Right parietal pleura	☐ Pleura	☐ Other
☐ Left parietal pleura	☐ Pericardium	☐ Not Specified
☐ Diaphragmatic pleura	☐ Peritoneum	

5. **Site of Metastasis:** ____
____

6. **Invasive Tumor Present?** (circle one)

Yes No Unknown Not applicable

*7. **Primary or Metastatic Tumor?** (circle one)

Primary Metastasis Not specified

*8. **Date of Resection or Biopsy:** __ __/__ __ __ __ (MM/YYYY)

9. **Tumor Size:**

Tumor size can be determined? (circle one) Yes No

Greatest dimension: __ __ **cm**

Additional dimension: __ __ **cm**

Maximum thickness: __ __ **cm**

*10. **Most Prominent Histological Type of Invasive Cancer:** (check one)

☐ Epithelial or epithelioid	☐ Biphasic	☐ Other
☐ Sarcomatoid	☐ Multicystic	☐ Not Specified
☐ Desmoplastic	☐ Papillary	☐ Unknown

*11. **Tumor Differentiation or Grade:** (check one)

☐ High	☐ Low	☐ Not specified
☐ Intermediate	☐ Indeterminate	☐ Not applicable

12. **Size of Largest Individual Nodule of Cancer:** __ . __ __ **cm**

*13. **Tumor Configuration:** (check one)

☐ Exophytic ☐ Papillary ☐ Diffuse

☐ Endophytic ☐ Cystic ☐ Localized
☐ Nodular ☐ Mixed ☐ Not Specified

14. Extent of Invasion: (check all that apply)

☐ Lung parenchyma	☐ Vertebra	☐ Diaphragm	☐ Other
☐ Endothoracic fascia	☐ Brachial plexus	☐ Peritoneum	☐ Cannot be determined
☐ Contralateral pleura	☐ Mediastinum	☐ Omentum	☐ Not applicable
☐ Soft tissue of chest wall	☐ Pericardium	☐ Abdominal viscera	
☐ Rib	☐ Myocardium	☐ Pelvic viscera	

15. Surgical Margin Involvement: (circle one)

Yes No Unknown Not applicable

16. Presence of Angiolymphatic Invasion: (circle one)

Yes No Unknown Not applicable

17. Extrapleural, Extraperitoneal or Extrapericardial Extension: (circle one)

Yes No Unknown Not applicable

18. Additional Pathologic Findings: (check all that apply)

☐ Ferruginous bodies	☐ Acute inflammation	☐ Changes of talc pleurodesis
☐ Pleural plaques	☐ Chronic inflammation	☐ Other
☐ Pulmonary interstitial fibrosis	☐ Mixed inflammation	☐ None identified

19. Histochemical Profile: ____
____
____

20. Immunohistochemical Profile: ____________________________________
____
____

21. Ultrastructural Findings: _______________________________________
____
____

22. General Comments Section on Resection/Biopsy: ______________________
____
____
____

<u>Neoplastic Paraffin Block Matrix:</u> (These are multiple entry fields. <u>Up to 4 blocks preferred</u>)

The Paraffin block matrix section will allow the collection of block details on cases that meet the minimum requirements for inclusion into the resource.

<u>NOTE</u>: Priorities for entering cases in resected mesothelioma block matrix.

- **These should assist the teams in picking the highest value blocks for the MVB archives.**
- **Since the matrix can include <u>up to 4 blocks</u> here are the recommendations for selection criteria:**
  1) **The *<u>first block</u>* should include the largest nodule of tumor (as specified by the CDE)**
  2) **The *<u>third and fourth blocks</u>* should include surgical margin involvement (SM) or angiolymphatic invasion (AL) (in that order of preference).**

NOTE: If SM, and AL do not occur, select the next largest area of tumor for 3 and 4 block of the matrix.

3) **The *second block* should include the second largest tumor.**

1. Block Matrix: Type(s) of Block(s) Available: (check one)

☐ Paraffin ☐ Frozen ☐ Both

2. Block Matrix: Accession Number (i.e. SP-91-645)**:**
__ __ __ - __ __ - __ __ __ __ __

3. Paraffin Block Matrix: Block Number (i.e. 1A, 12AA)**:** __ __ __ __

4. Paraffin Block Matrix: Most Prominent Histological Type: (check one)

☐ Epithelial or epithelioid	☐ Biphasic	☐ Other
☐ Sarcomatoid	☐ Multicystic	☐ Not Specified
☐ Desmoplastic	☐ Papillary	☐ Unknown

5. Paraffin Block Matrix: Size of Largest Individual Nodule of Cancer:
__ . __ __ cm

6. Paraffin Block Matrix: Presence of Positive Surgical Margin (SM): (circle one)
Yes No Unknown Not applicable

7. Paraffin Block Matrix: Presence of Angiolymphatic Invasion (AL): (circle one)
Yes No Unknown Not applicable

8. Paraffin Block Matrix: Block Comments: ______________________________
____
____
____

Non-Neoplastic Paraffin Block Matrix:

Try to include at least one block of normal lung if possible (two blocks are preferred). If it is not possible to find a completely normal block then include one with minimal amounts of mesothelioma tissue.

1. Paraffin Resection Specimen Non-Neoplastic Block #1 (i.e. 1A, 12AA)**:**
__ __ __ __

2. Paraffin Resection Specimen Non-Neoplastic Block #2 (i.e. 1A, 12AA)**:**
__ __ __ __

3. General Comments on Paraffin Blocks: ______________________________
____
____
____

Neoplastic Frozen Bulk Block Matrix: **(These are multiple entry fields. Up to 4 blocks preferred)**

Same as above.

NOTE: This section is for the FROZEN BLOCK matrix.

It is very possible that some (or all) of the frozen blocks in the matrix will also have paraffin tissue. When this happens, indicate this in data element [Type of Block(s) available]. On occasion there may be some blocks that are only frozen (site dependent) - also indicate this in the aforementioned data items.

1. **Block Matrix: Type(s) of Block(s) Available:** (check one)

☐ **Paraffin** ☐ **Frozen** ☐ **Both**

2. **Frozen Tissue: Warm Ischemic Time: __ __ __ minutes**
3. **Frozen Block Matrix: Block Number** (i.e. 1A, 12AA)**: __ __ __ __**
4. **Frozen Block Matrix: Most Prominent Histological Type:** (check one)

☐ **Epithelial or epithelioid**	☐ **Biphasic**	☐ **Other**
☐ **Sarcomatoid**	☐ **Multicystic**	☐ **Not Specified**
☐ **Desmoplastic**	☐ **Papillary**	☐ **Unknown**

5. **Frozen Block Matrix: Size of Largest Individual Nodule of Cancer: __ . __ __ cm**
6. **Frozen Block Matrix: Presence of Positive Surgical Margin (SM):** (circle one)
   Yes No Unknown Not applicable
7. **Frozen Block Matrix: Presence of Angiolymphatic Invasion (AL):** (circle one)
   Yes No Unknown Not applicable
8. **Frozen Block Matrix: Block Comments: ________________________________**
   ____
   ____
   ____

Non-neoplastic Bulk Frozen Blocks:

Try to include at least one block of normal lung if possible (two blocks are preferred). If it is not possible to find a completely normal block then include one with minimal amounts of mesothelioma tissue.

1. **Frozen Resection Specimen Non-Neoplastic Block #1** (i.e. 1A, 12AA)**: __ __ __ __**
2. **Frozen Resection Specimen Non-Neoplastic Block #2** (i.e. 1A, 12AA)**: __ __ __ __**
3. **General Comments on Frozen Blocks: ________________________________**
   ____
   ____
   ____

Biopsy Block Matrix:

The biopsy block matrix section will allow the collection of block details on biopsy samples that meet the minimum requirements for inclusion into the Resource.

- **For biopsy cases, the concern is to give the reviewers some guidance on how many blocks to submit and to clarify what happens when <u>there is only one block with tumor</u>. In these cases it will be recommended that the institution cut 3 to 5 blanks for their own diagnostic purposes and then make the residual material available to the MVB archive.**
- **A matrix block will be set up and leave the number of blocks submitted to the reviewing pathologist, encouraging them to submit as many blocks as possible on a biopsy to a maximum of 5. There will be no particular order for entering blocks into the biopsy matrix.**

- **The recommended criteria for biopsy will be as follows:**
  1) **Can include one block or more (up to 5)**
  2) **Must at least include one neoplastic block and classify according to the biopsy matrix.**

1. Biopsy Block Matrix: Block Number (i.e. 1A, 12AA)**: __ __ __ __**

2. Biopsy Block Matrix: Most Prominent Histological Type: (check one)

☐ Epithelial or epithelioid	☐ Biphasic	☐ Other
☐ Sarcomatoid	☐ Multicystic	☐ Not Specified
☐ Desmoplastic	☐ Papillary	☐ Unknown

3. Biopsy Block Matrix: Grade: (check one)

☐ High	☐ Low	☐ Not specified
☐ Intermediate	☐ Indeterminate	☐ Not applicable

4. Biopsy Block Matrix: Size of Largest Individual Nodule of Cancer: __ . __ __ cm

5. Biopsy Block Matrix: Presence of Angiolymphatic Invasion (AL): (circle one)
Yes No Unknown Not applicable

6. Biopsy Block Matrix: Block Comments: ________________________________
____
____
____

Regional Lymph Node Status at Time of Resection:

Include the lymph node status at the time of the resection.

-If there is regional lymph node exploration prior or equal to the resection date, but after the initial diagnostic biopsy, then enter values here (in the Lymph Node Block Matrix section).

-Regional lymph nodes removed <u>after</u> the resection of tumor or distant lymph nodes should be enter in the Metastatic Tissue Block Matrix section.

1. Date of Regional Lymph Node Resection: __ __ /__ __ __ __ (MM/YYYY)

2. Number of Lymph Nodes Examined: __ __ __

3. Number of Lymph Nodes Positive: __ __ __

4. Lymph Node Block Matrix: Non-Neoplastic Block Number (i.e. 1A, 12AA)**:**
__ __ __ __

5. Lymph Node Block Matrix: Neoplastic Block Number (i.e. 1A, 12AA)**:**
__ __ __ __

6. Lymph Node Block Matrix: Size of Largest Individual Nodule of Cancer: __ . __ __ cm

7. Lymph Node Block Matrix: Presence of Extracapsular Extension (ECE):
(circle one) Yes No Unknown

8. General Comments Section for Lymph Nodes:___________________________
____
____
____

Recurrence / Metastasis Data:

Metastatic Tissue Block Matrix for cases that have tissue available through the resource from anatomical sites that show recurrence / metastasis of mesothelioma.

Metastatic Tissue Block Matrix:

-Enter as many blocks available (Three blocks are preferred).
-If multiple metastatic sites are present, then enter at least 1 block from each site.
-Try to include at least one block of normal tissue from the same site if possible. If it is not possible to find a completely normal block, then include one with minimal amounts of tumor.

1. Metastatic Block Matrix: Type(s) of Block(s) Available: (check one)

☐ Paraffin ☐ Frozen ☐ Both

2. Date of Metastatic Block Matrix: __ __ /__ __ __ __ (MM / YYYY)

3. Metastatic Block Matrix: Block Number (i.e. 1A, 12AA)**:** __ __ __ __

4. Metastatic Block Matrix: Specimen Source: (check one)

☐ Resection ☐ Biopsy ☐ Both ☐ Not Specified

5. Biopsy Block Matrix: Most Prominent Histological Type: (check one)

☐ Epithelial or epithelioid	☐ Biphasic	☐ Other
☐ Sarcomatoid	☐ Multicystic	☐ Not Specified
☐ Desmoplastic	☐ Papillary	☐ Unknown

6. Metastatic Block Matrix: Size of Largest Individual Nodule of Invasive Cancer: __ . __ __ cm

7. Metastatic Block Matrix: Presence of Therapy Effects: (check all that apply)

☐ Surgery	☐ Immunotherapy	☐ Watchful expectany
☐ Radiation therapy	☐ Brachytherapy	☐ Other
☐ Chemotherapy	☐ Cryotherapy	☐ None
☐ Hormone therapy	☐ External beam	

8. Metastatic Block Matrix: Non-Neoplastic Block Number (i.e. 1A, 12AA)**:** __ __ __ __

9. General Comments Section for Metastatic Block Matrix:__________________
____
____
____

GENOTYPES DATA

1. Genotype Data Available: (check one)

☐ Yes ☐ No ☐ Unknown ☐ Not assessed

2. GSTM1 Genotype: (check one)

☐ Functional (0) ☐ Null (2)

3. GSTTM1 Genotype: (check one)

☐ Functional (0) ☐ Null (2)

4. CYP1A1 Genotype: (check one)

☐ Homozygous wild-type (0) ☐ Heterozygous (1) ☐ Homozygous variant (2)

5. NAT2 Genotype: (check one)

☐ Slow (0) ☐ Fast (1)

6. EPHX1 Genotype: (check one)

☐ High (0) ☐ Intermediate (1) ☐ Low (2)

7. EPHX3 Genotype: (check one)

☐ Homozygous wild-type (0) ☐ Heterozygous (1) ☐ Homozygous variant (2)

8. EPHX4 Genotype: (check one)

☐ Homozygous wild-type (0) ☐ Heterozygous (1) ☐ Homozygous variant (2)

STAGING

Pathological and Clinical Staging will be based on using the <u>AJCC Manual for Staging of Cancer</u>.

AJCC Manual for Staging of Cancer:

AJCC Manual Edition Number: (check one)

☐ 6th	☐ 4th	☐ 2nd	☐ Not applicable
☐ 5th	☐ 3rd	☐ 1st	☐ Unknown

Pathological Staging:

1. pT Stage: (check one)

☐ pTX	☐ pT1b	☐ pT4
☐ pT0	☐ pT2	☐ Not applicable
☐ pT1a	☐ pT3	☐ Unknown

AJCC (6th Ed.) Staging for Thoracic Mesothelioma	
**pTX**	Primary tumor cannot be assessed
**pT0**	No evidence of primary tumor
**pT1**	Tumor involves ipsilateral parietal pleura, with or without focal involvement of visceral pleura
**pT1a**	Tumor involves ipsilateral parietal (mediastinal, diaphragmatic) pleura. No involvement of the visceral pleura
**pT1b**	Tumor involves ipsilateral parietal (mediastinal, diaphragmatic) pleura, with focal involvement of the visceral pleura
**pT2**	Tumor involves any of the ipsilateral pleural surfaces with at least 1 of the following: confluent visceral pleural tumor (including fissure), invasion of diaphragmatic muscle, invasion of lung parenchyma
**pT3**	Tumor involves any of the ipsilateral pleural surfaces, with at least 1 of the following: invasion of the endothoracic fascia, invasion into mediastinal fat, solitary focus of tumor invading the soft tissues of the chest wall, non-transmural involvement of the pericardium
**pT4**	Tumor involves any of the ipsilateral pleural surfaces, with at least 1 of the following: diffuse or multifocal invasion of soft tissues of the chest wall, any involvement of rib, invasion through the diaphragm to the peritoneum, invasion of any mediastinal organ(s), direct extension to the contralateral pleura, invasion into the spine, extension to the internal surface of the pericardium, pericardial effusion with positive cytology, invasion of the myocardium, invasion of the brachial plexus

2. pN Stage: (check one)

☐ pNX	☐ pN2	☐ Unknown
☐ pN0	☐ pN3	
☐ pN1	☐ Not applicable	

AJCC (6th Ed.) Staging for Thoracic Mesothelioma	
**pNX**	Regional lymph nodes cannot be assessed
**pN0**	No regional lymph node metastases
**pN1**	Metastases in the ipsilateral bronchopulmonary and/or hilar lymph node(s)
**pN2**	Metastases in the subcarinal lymph node(s) and/or the ipsilateral internal mammary or mediastinal lymph node(s)
**pN3**	Metastases in the contralateral mediastinal, internal mammary, or hilar lymph node(s) and/or the ipsilateral or contralateral supraclavicular or scalene lymph node(s)

3. pM Stage: (check one)

☐ pMX ☐ pM1 ☐ Unknown

☐ pM0 ☐ Not applicable

AJCC (6th Ed.) Staging	
pMX	Presence of distant metastasis cannot be assessed
pM0	No known distant metastasis
pM1	Distant metastasis

Clinical Staging:

1. cT Stage: (check one)

☐ cTX ☐ cT1 ☐ cT3 ☐ Not applicable
☐ cT0 ☐ cT2 ☐ cT4 ☐ Unknown

AJCC (6th Ed.) Staging for Thoracic Mesothelioma	
cTX	Primary tumor cannot be assessed
cT0	No evidence of primary tumor
cT1	Tumor limited to ipsilateral parietal and/or visceral pleura
cT2	Tumor invades any of the following: ipsilateral lung, endothoracic facia, diaphram, or pericardium
cT3	Tumor invades any of the following: ipsilateral chest wall muscle, ribs, or mediastinal organs or tissues
cT4	Tumor directly extends to any of the following: contralateral pleura, lung, peritoneum, intra-abdominal organs or cervical tissues

2. cN Stage: (check one)

☐ cNX ☐ cN1 ☐ cN3 ☐ Unknown
☐ cN0 ☐ cN2 ☐ Not applicable

AJCC (6th Ed.) Staging for Thoracic Mesothelioma	
cNX	Regional lymph nodes cannot be assessed
cN0	No regional lymph node metastasis
cN1	Metastasis in ipsilateral peribronchial and/or ipsilateral hilar lymph nodes, including direct extension
cN2	Metastasis in ipsilateral mediastinal and/or subcarinal lymph node(s)
cN3	Metastasis in contralateral mediastinal, contralateral hilar, ipsilateral or contralateral scalene, or supraclavicular lymph node(s)

3. cM Stage: (check one)

☐ cMX ☐ cM1 ☐ Unknown
☐ cM0 ☐ Not applicable

AJCC (6th Ed.) Staging	
cMX	Presence of distant metastasis cannot be assessed
cM0	No distant metastasis
cM1	Distant metastasis

4. General Staging Comments: ____
____
____
____

THERAPY RELATED DATA

Record the patient's treatment history.

***1. Therapy matrix: Type of Therapy: (check all that apply)**

☐ Surgery	☐ Immunotherapy	☐ Watchful expectany
☐ Radiation therapy	☐ Brachytherapy	☐ Other
☐ Chemotherapy	☐ Cryotherapy	☐ None
☐ Hormone therapy	☐ External beam	

2. Therapy matrix: Therapy Start Date: __ __ / __ __ __ __ (MM / YYYY)

3. Presence of pleural effusion: (circle one) Yes No Unknown

4. Presence of ascites: (circle one) Yes No Unknown

5. Comments on Therapy Response: ____________________________________
____
____
____

6. General Overall Comments on Therapy: _______________________________
____
____
____

CLINICALLY VERIFIED TISSUE RECURRENCE / METASTASIS DATA

These cases are those that do NOT have tissue blocks available, but are known clinically to have a recurrence/metastasis.

NOTE: Verification for clinical recurrence can be via radiology imaging, biopsy/resection, surgery, or cancer registry. However, a clinician's note indicating recurrence in a specific distant site would be sufficient.

1. Date of Tissue Recuurence / Metastasis: Distant Site #1: __ __ / __ __ __ __
(MM / YYYY)

2. Tissue Recurrence / Metastasis: Distant Site #1: (check one)

- ☐ Adrenal
- ☐ Anus
- ☐ Appendix
- ☐ Bones
- ☐ Breast
- ☐ Brain & CNS
- ☐ Colon
- ☐ Cervix Uteri
- ☐ Corpus Uteri
- ☐ Esophagus
- ☐ Gallbladder
- ☐ Head and Neck
- ☐ Kidney, Renal Pelvis, Ureter
- ☐ Larynx
- ☐ Leukemia
- ☐ Liver
- ☐ Lung
- ☐ Lymph node
- ☐ Mesothelioma
- ☐ Ovary
- ☐ Oral Cavity
- ☐ Pancreatic
- ☐ Parotid & Other Glands
- ☐ Pharynx
- ☐ Larynx
- ☐ Pleura
- ☐ Prostate
- ☐ Rectosigmoid
- ☐ Rectum
- ☐ Skin
- ☐ Spleen
- ☐ Stomach
- ☐ Testis
- ☐ Thyroid
- ☐ Urinary Bladder
- ☐ Other, NOS
- ☐ Unknown
- ☐ None

3. Tissue Recurrence / Metastasis: Distant Site #2: (check one)

- ☐ Adrenal
- ☐ Anus
- ☐ Appendix
- ☐ Bones
- ☐ Breast
- ☐ Brain & CNS
- ☐ Colon
- ☐ Cervix Uteri
- ☐ Corpus Uteri
- ☐ Esophagus
- ☐ Gallbladder
- ☐ Head and Neck
- ☐ Kidney, Renal Pelvis, Ureter
- ☐ Larynx
- ☐ Leukemia
- ☐ Liver
- ☐ Lung
- ☐ Lymph node
- ☐ Mesothelioma
- ☐ Ovary
- ☐ Oral Cavity
- ☐ Pancreatic
- ☐ Parotid & Other Glands
- ☐ Pharynx
- ☐ Larynx
- ☐ Pleura
- ☐ Prostate
- ☐ Rectosigmoid
- ☐ Rectum
- ☐ Skin
- ☐ Spleen
- ☐ Stomach
- ☐ Testis
- ☐ Thyroid
- ☐ Urinary Bladder
- ☐ Other, NOS
- ☐ Unknown
- ☐ None

4. Tissue Recurrence / Metastasis: Distant Site #3: (check one)

- ☐ Adrenal
- ☐ Anus
- ☐ Appendix
- ☐ Bones
- ☐ Breast
- ☐ Brain & CNS
- ☐ Colon
- ☐ Cervix Uteri
- ☐ Corpus Uteri
- ☐ Larynx
- ☐ Leukemia
- ☐ Liver
- ☐ Lung
- ☐ Lymph node
- ☐ Mesothelioma
- ☐ Ovary
- ☐ Oral Cavity
- ☐ Pancreatic
- ☐ Prostate
- ☐ Rectosigmoid
- ☐ Rectum
- ☐ Skin
- ☐ Spleen
- ☐ Stomach
- ☐ Testis
- ☐ Thyroid
- ☐ Urinary Bladder

- ☐ **Esophagus**
- ☐ **Gallbladder**
- ☐ **Head and Neck**
- ☐ **Kidney, Renal Pelvis, Ureter**
- ☐ **Parotid & Other Glands**
- ☐ **Pharynx**
- ☐ **Larynx**
- ☐ **Pleura**
- ☐ **Other, NOS**
- ☐ **Unknown**
- ☐ **None**

5. General Comments for Clinically Verified Tissue Recurrence / Metastasis:____

____

____

____

____

VITAL STATUS / FOLLOW-UP DATA

Record the patient's vital status and most recent follow up date.

***1. Vital Status:** (check one)

☐ **Alive** ☐ **Dead** ☐ **Unknown**

***2. Cancer Status:** (check one)

☐ **No evidence of cancer** ☐ **Evidence of cancer** ☐ **Unknown**

***3. Date Last Known Alive: __ __ / __ __ __ __ (MM / YYYY)**

4. Date of Death: __ __ / __ __ __ __ (MM / YYYY)

5. Cause of Death: (check one)

☐ **Directly** ☐ **Indirectly** ☐ **Not caused by cancer** ☐ **Unknown**

6. Final Comments: ____

____

____

____
