# Supplementary material for: The development and deployment of Common Data Elements for tissue banks for translational research in cancer – An emerging standard based approach for the Mesothelioma Virtual Tissue Bank
Source: BMC Cancer. 2008 Apr 8;8:91. doi: 10.1186/1471-2407-8-91 (PMC2329649; doi:10.1186/1471-2407-8-91)
Supplement: Additional file 7 — Tissue Bank Workflow for MVB across the institutions. [file 1471-2407-8-91-S7.doc]

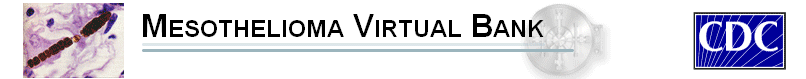


**Tissue Bank Workflow for MVB ACROSS THE INSTITUTIONS**

# **The Centers for Disease Control and Prevention (CDC) funded the Mesothelioma Grant**

**Central Cancer Registry pooled all Mesothelioma cases within the UPMC Health System (Presbyterian, Shadyside & Magee-Womens Hospitals), and sent the list of cases to the Biomedical Informatics Team**

**Biomedical Informatics Team queried the CoPath system to get the associated accession numbers, and generated a spreadsheet of the potential cases**

**Spreadsheet given to the Tissue Bank Team, who then determines the location of the blocks/slides through CoPath (On-site, Iron Mountain off-site storage, physician offices, or law offices)**

**Cases put together by Tissue Bankers, and logged onto spreadsheet**

**Iron Mountain processed request, and sent cases to Tissue Bank**

**Cases sent to MVB Team (all participating Institutions and the Central Data Center)) for review**

**Cases listed on Iron Mountain request forms, which are given to Pathology Department for faxing to Iron Mountain**

**Cases pulled by Tissue Bankers, and the number of blocks/ slides documented on the spreadsheet**

**Off-Site**

**On-Site**

**Participating Institutions (New York University and University of Pennsylvania) sends the multimodal datasets as per the attributes and valid values in the form of excel workbook to the Biomedical Informatics Team**
