# Supplementary material for: The development and deployment of Common Data Elements for tissue banks for translational research in cancer – An emerging standard based approach for the Mesothelioma Virtual Tissue Bank
Source: BMC Cancer. 2008 Apr 8;8:91. doi: 10.1186/1471-2407-8-91 (PMC2329649; doi:10.1186/1471-2407-8-91)
Supplement: Additional file 8 — CDC MVB Tissue Request Work Flow Chart. [file 1471-2407-8-91-S8.doc]

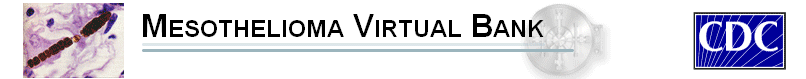


**CDC MVB Tissue Request Work Flow Chart**

**The Primary Investigator (PI) or researcher browses and queries the publicly available MBV web site (**[**www.mesotissue.org**](http://www.mesotissue.org/)**) to determine potential cases in the data base that meet criteria of his/her research project.**

**The PI or researcher then sends his/her inquiry to MVB Project Coordinator (PC) (Department of Biomedical Informatics [DBMI], University of Pittsburgh School Medicine, Pittsburgh, PA) via e-mail through the MVB web site (most often), telephone, or letter (rarely) to request tissue.**

**1 – 2**

**Days**

**PC responds to PI or researcher by providing information on how to submit Letter of Intent (LOI) (LOI is required before PI or researcher submits a complete application). If the MVB is not appropriate for the PI or researcher, then he/she would be directed to other possible resources, if known.**

**10 Days**

**PI or researcher completes LOI, which includes description of the scientific aim of his/her study and the number of samples being requested.**

**1 Month**

**Approved**

**Disapproved**

**Once LOI received by PC, it is numbered (e.g. L-####) and forwarded to Research Evaluation Panel (REP) who reviews LOI to determine the scientific merit and feasibility of the project. Notification is sent to PI or researcher acknowledging receipt of LOI and its assigned number.**

**LOI sent back to PI or researcher requesting more detail and/or answers to questions raised by REP members.**

**REP statistician does analysis of approved LOI. An approval notice is sent to PI or researcher.**

**2 Weeks**

**PI or researcher resubmits LOI to REP with additional information.**

**A REP member is assigned to guide PI or researcher on submission of full application for the Bank. Fee structure will be discussed.**

**1 Day**

**PI or researcher sends full application to PC for submission to Coordinating Committee.**

**REP reviews the new information.**

**2 Weeks**

**Approved**

**Disapproved**

**1 Week**

**PI or researcher resubmits full application with additional/corrected information.**

**Approval notification sent to PI or researcher with new Research Request Number (LOI number [L-####] with preceding L replaced with an R [R-####]).**

**1 Day**

**PI or researcher sends to DBMI a Letter of Agreement to Pay signed by himself/herself and an Institutional official.**

**1 Week**

**DBMI post-doctoral fellows select cases meeting research study criteria, create a spreadsheet, and submit it and PI or researcher contact information to Health Sciences Tissue Bank (HSTB), Department of Pathology, University of Pittsburgh. Material Transfer Agreement (MTA) is initiated by HSTB staff and then sent to Grants Administration within Department of Pathology (University of Pittsburgh).**

**2 Weeks**

**up to**

**4 Weeks**

**University of Pittsburgh Office of Human Research approves MTA.**

**Requester’s institutional Office of Human Research equivalent approves MTA.**

**1 Month**

**HSTB assembles requested material and prepares it for shipping to destination. DBMI prepares spreadsheet containing required de-identified annotated data.**

**1 Day**

**1 Day**

**DBMI sends invoice for total cost of all research material.**

**DBMI & HSTB inform PI or researcher (or designated tissue recipient) that material and annotated data have been shipped/sent.**

**The appropriate investigative staff receives research material along with Customer Satisfaction Survey (i.e., receipt of tissue, timeliness of shipment, quality of material) within 90 days of final shipment of research material.**

**Approximate**

**Total time =**

**5 Months**

**Note: LOI disapproval**

**adds 1 month**

**🞎 Researcher 🞎 DBMI 🞎 HSTB 🞎 Office of Human Research**
